# Quantum Computational Investigation of (*E*)-1-(4-methoxyphenyl)-5-methyl-*N*′-(3-phenoxybenzylidene)-1*H*-1,2,3-triazole-4-carbohydrazide

**DOI:** 10.3390/molecules27072193

**Published:** 2022-03-28

**Authors:** Halil Gökce, Fatih Şen, Yusuf Sert, Bakr F. Abdel-Wahab, Benson M. Kariuki, Gamal A. El-Hiti

**Affiliations:** 1 Vocational School of Health Services, Giresun University, 28200 Giresun, Turkey; 2 Sorgun Vocational School, Bozok University, 66700 Yozgat, Turkey; fatihsen55@gmail.com (F.Ş.); yusufsert1984@gmail.com (Y.S.); 3 Department of Physics, Faculty of Art & Science, Bozok University, 66900 Yozgat, Turkey; 4 Applied Organic Chemistry Department, National Research Centre, Dokki, Giza 12622, Egypt; bakrfatehy@yahoo.com; 5 School of Chemistry, Cardiff University, Main Building, Park Place, Cardiff CF10 3AT, UK; kariukib@cardiff.ac.uk; 6 Department of Optometry, College of Applied Medical Sciences, King Saud University, Riyadh 11433, Saudi Arabia

**Keywords:** DFT, Hirshfeld surface analysis, 1*H*-1,2,3-triazole-4-carbohydrazide, molecular docking, drug-likeness

## Abstract

The title compound was synthesized and structurally characterized. Theoretical IR, NMR (with the GIAO technique), UV, and nonlinear optical properties (NLO) in four different solvents were calculated for the compound. The calculated HOMO–LUMO energies using time-dependent (TD) DFT revealed that charge transfer occurs within the molecule, and probable transitions in the four solvents were identified. The in silico absorption, distribution, metabolism, and excretion (ADME) analysis was performed in order to determine some physicochemical, lipophilicity, water solubility, pharmacokinetics, drug-likeness, and medicinal properties of the molecule. Finally, molecular docking calculation was performed, and the results were evaluated in detail.

## 1. Introduction

Due to their biological activities, 1,2,3-triazoles are associated with many medicinal applications [1,2,3,4]. They act as antidiabetic [5], anti-inflammatory [6], antifungal [7], antibacterial [7,8,9], and antiviral [10] agents. Compounds containing a pendant 1,2,3-triazole ring system are active ingredients in medications such as tazobactam and cefatrizine [11,12]. Other triazole derivatives are also effective, as illustrated by antitumoral activity of carboxyamidotriazole and application of rufinamide as an antiepileptic drug in the treatment of partial seizures [13,14].

Based on their wide range of biological activities, the development of a variety of synthetic routes is worthwhile, and generation of new triazole derivatives is beneficial. The synthesis of 1,2,3-triazoles, for example, involves 1,3-dipolar cycloaddition in the presence of copper iodide [15], coupling of sodium azide and *N*-tosylhydrazones in the presence of iodine [16], the reaction of sodium azide and nitroolefin in the presence of Amberlyst 15 [17], the reaction of sodium azide and alkenyl bromides in the presence of a palladium catalyst [18], and the reaction of aryl azides and allenylindium bromide in the presence of *n*-butylamine [19].

The investigation reported here involves the synthesis, experimental and theoretical vibrational analysis of (*E*)-1-(4-methoxyphenyl)-5-methyl-*N*′-(3-phenoxybenzylidene)-1*H*-1,2,3-triazole-4-carbohydrazide (**2**) as a continuation of our work in the field [20,21,22,23]. Of particular interest is the agreement between the theoretical results obtained and the experimental data. Computational approaches have become increasingly popular in recent years for elucidating the molecular-level properties of molecules. The behavior of molecules can thus be predicted without the need for experimental procedures. In the fields of pharmacy, pharmacology, and materials engineering, DFT, ab initio molecular mechanics, and various semiexperimental approaches are frequently utilized in the study of molecular characteristics. For example, molecular docking was used recently to investigate the reactivity and potential use of niclosamide and 1-ethylpiperazine-1,4-diium *bis*(nitrate) for the treatment of COVID-19 [24,25].

A detailed study of the chemical activity of **2** was carried out utilizing theoretical computational chemistry. The Hirshfeld surface analysis approach was used to investigate interactions between molecules, the percentage contribution of atom-to-atom interactions, fingerprint determination, and total surface mapping. The TDDFT approach was applied with various solvents to better understand the effect of solvent electrical properties on the UV spectra. Additionally, the aim of the in silico analysis in the current study was to assess the drug-likeness profile, investigation of ADME properties, computer-based computational biological activity prediction, and the molecular docking for compound 2. The compound contains the 1,2,3-triazole moiety and is expected to be biologically active with useful medicinal applications [1,2,3,4].

## 2. Results and Discussion

### 2.1. Crystal Structure

**Figure 1:**
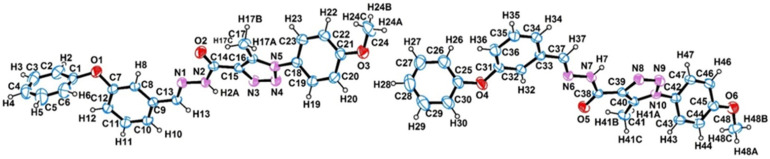
The two molecules of the asymmetric unit of **2** with the atom-labeling scheme. Displacement ellipsoids are drawn at the 50% probability level.

The ORTEP representation of the asymmetric unit of compound **2** with atomic numbering is shown in Figure 1. Compound **2** crystallizes in the triclinic system with space group *P*$\bar{1}$ and cell dimensions a = 6.6452 (5) Å, b = 7.4466 (5) Å, c = 43.403 (2) Å, α = 86.250° (4), β = 89.193° (5), and γ = 80.484° (6), with four molecules (Z) in the unit cell. Hence, there are two independent molecules in the asymmetric unit. For the 1,2,3-triazole group, the average C=C, N=N, and N–N bond lengths for the two independent molecules are 1.368 Å, 1.3045 Å, and 1.37 Å, respectively, and the value for the N–N–N angle is 104.715°. These bond lengths and the angle are consistent with those reported [26,27,28]. It should be noted that the presence of hydrogen bonds has an influence on the vibration modes of some functional groups such as the OH and NH moieties which is consistent with the literature [29]. The 1,2,3-triazole rings are planar, with maximum deviation from the plane of only 0.0063 Å (N8, N9, N10, C40, C39) and 0.0049 Å (N3, N4, N5, C16, C15). The Schiff base (N6=C37 and N1=C13) average bond length is 1.2855 Å, which is close to the corresponding values previously reported as 1.272 Å [30], 1.269 Å [31], and 1.283 Å [32]. The dihedral angles between planes A (C1/2/3/4/5/6), B (C7/8/9/10/11/12), C (1,2,3-triazole ring; N3, N4, N5, C16, C15), and D (C18/19/20/21/22/23) are 85.320° (A/B), 65.823° (A/C), 71.638° (A/D), 19.929° (B/C), 26.276° (B/D), and 43.343° (C/D).

**Figure 2:**
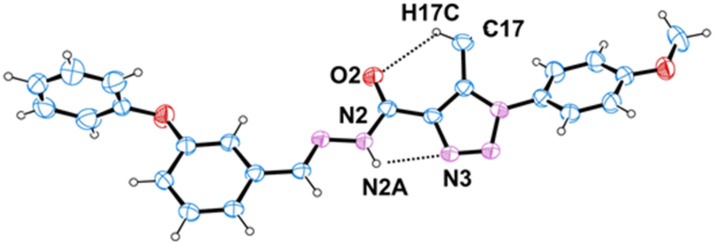
Intramolecular hydrogen bonding of **2**.

Parst [33] analysis indicates that there are potentially two intramolecular and two intermolecular interactions for each of the two molecules in the asymmetric unit. The details are given in Table 1. The C17–H17C···O2 and N2–H2A···N3 intramolecular hydrogen bonds forming S(6) and S(5) ring motifs, respectively, for one molecule are illustrated in Figure 2.

**Figure 3:**
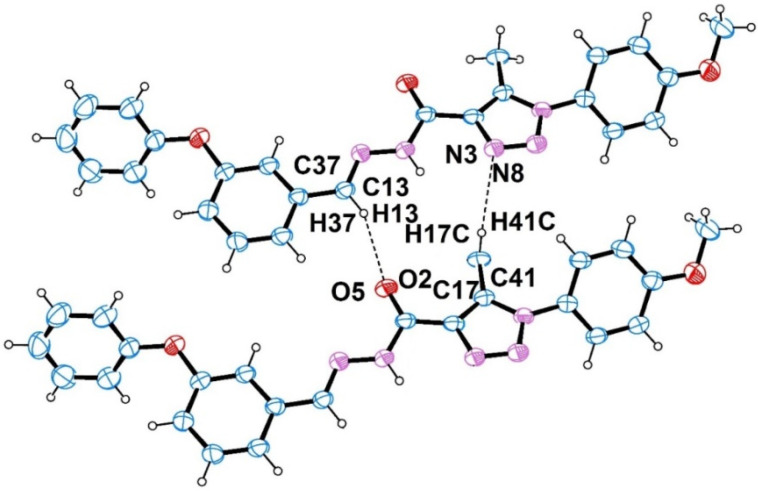
The graphical representation showing hydrogen bonding and motif ($R_{2}^{2}\left(13\right)$) in the crystal structure of **2**. Atoms O2, H13, C13, C17, N3, and H17C refer to one pair of molecules, whereas O5, H37, C37, C41, H41C, and N8 refer to another pair.

For one molecule, atom O2 acts as an acceptor, via atom H13, to atom C13 of a neighboring molecule. C17 acts as a donor to N3 of the same neighboring molecule via atom H17C to form a ($R_{2}^{2}\left(13\right)$) motif. For the second molecule, atom O5 acts as an acceptor, via atom H37, to atom C37, and C41 acts as a donor, via H41C, to N8 in a neighboring molecule to form a ($R_{2}^{2}\left(13\right)$) motif (Figure 3).

**Figure 4:**
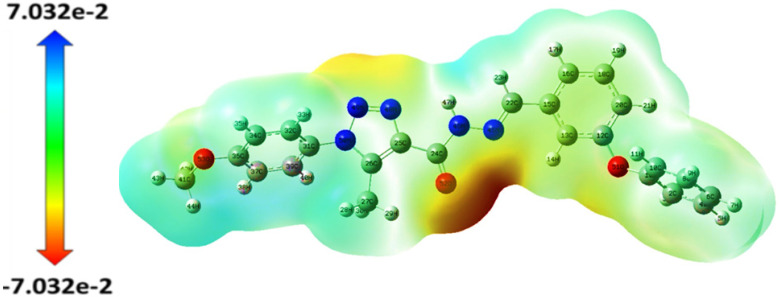
The MEPS for the optimized geometry of **2** (DFT/B3LYP/6-311++G(d,p)).

The computational quantum-mechanical modeling calculations were carried out using the B3LYP/6-311++G(d,p) level of the density functional theory (DFT) method. For modeling, the initial geometry of compound **2** was obtained from the crystallographic information file (CIF). Optimization was carried out by default spin, solvent-free on the ground state. The GaussView (ball and bond type) drawing for the molecular structure is presented in Figure 4. The electronic structure parameters for the theoretical molecule can be summarized as −1426.94224532 a.u. E(RB3LYP), 4.6382 Debye dipole moment, 106.731 Cal/mol/K heat capacity, and 196.914 Cal/mol/K entropy.

Some selected structural parameters revealed from the X-ray diffraction and calculated by the DFT/B3LYP/6-311++G(d,p) level are listed in Table 2. The computed R^2^ values are 0.9722 (y = 1.0616x − 0.0825) for the bond lengths, 0.9387 (y = 0.9279x + 9.601) for the bond angles, and 0.9907 (y = 1.0041x − 2.6247) for the torsion angles at the 6-311++G(d,p) level. The optimized geometry of the 1,2,3-triazole ring shows N–N and N=N bond lengths, calculated as 1.36720 and 1.29101 Å, respectively; they are essentially the same as those obtained from X-ray data (average = 1.37 and 1.3045 Å, respectively). The 1,2,3-triazole is quite planar as can be seen from the torsion angles (0.04213°, 0.28928°, –0.48051°, 0.49547°, and –0.34998°). At the DFT/B3LYP/6-311++g(d,p) level, the calculated D–H, H–A, D–A lengths and the D–H···A angle for the intramolecular bonding are 1.01912 Å, 2.26559 Å, 2.74092 Å, and 106.88299°, respectively, for the N2–H2*A*···N3 and N7–H7···N8 contacts. The corresponding angles are 1.08862 Å, 2.47608 Å, 3.14371 Å, and 118.43322°, respectively, for C17–H17*B*···O2 and C41–H41*B*···O5.

### 2.2. Surface Studies

#### 2.2.1. MEP Surface

The molecular electrostatic potential (MEP) map surface illustrates the three-dimensional electrostatic potential distributions of molecules. The regional electrostatic potential is indicated by the color of the surface. The electrophilic and nucleophilic centers of a molecule can be evaluated with the aid of colored regions. Green zones have zero potential, blue zones are electron-poor, with most positive electrostatic potential, and are nucleophilic centers, whereas red zones are electron-rich, with most electronegative electrostatic potential, and are electrophilic centers.

To assess the reactive centers for electrophilic and nucleophilic attack for compound **2**, the molecular electrostatic potential surface (MEPS) was calculated with Gaussian 09W [34] and viewed with the Gauss-View 5.0 [35] software using the Gaussian checkpoint file (*.chk). The color code of the map was in the range between −7.032e−2 a.u. (red) and 7.032e−2 a.u. (blue). In Figure 4, the red zones are on atoms O1, O2, N1, N3, and N4. The regions around these atoms are electron-rich and can be considered as electrophilic zones. Additionally, the result confirmed the disposition to form contacts as demonstrated by intramolecular N2–H2A···N3 and C17–H17B···O2 and intermolecular C13–H13···O2 and C17–H17C···N3 hydrogen bonding (Table 1).

#### 2.2.2. Hirshfeld Surface

**Figure 5:**
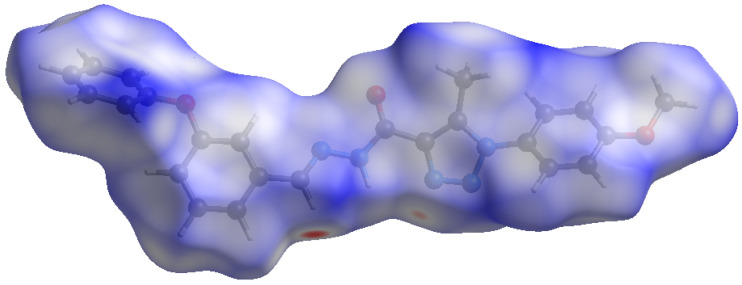
The Hirshfeld surface of a molecule of **2** mapped with d_norm_.

The Hirshfeld surface mapped with d_norm_ and fingerprint plots mapped over d_norm_ were generated with the CrystalExplorer 21.5 [36,37] software using the CIF. The surface analysis was carried out for a single molecule in the asymmetric unit of **2**. The Hirshfeld surface mapped with d_norm_ (Figure 5) with a fixed color scale of −0.2197 (red) to 1.4273 Å (blue) indicated a molecular volume of 518 Å^3^, a surface area of 481.47 Å^2^, with 0.648 globularity and 0.632 asphericity.

There are five red spots on the d_norm_ surface. These spots are indicators of short contacts such as hydrogen bonding interactions. The spots are on the O1, O2, N3, H13, and H17C atoms (Figure 5) and are consistent with the intermolecular interactions given in Table 1.

**Figure 6:**
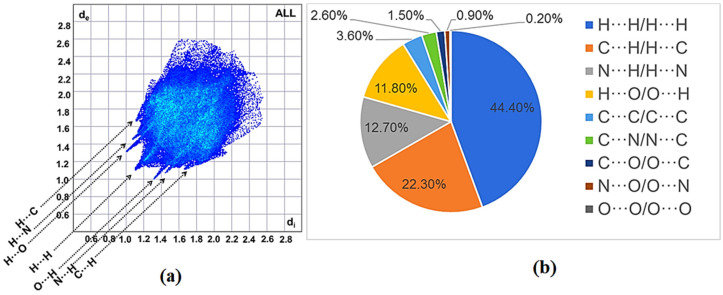
(**a**) The fingerprint plot of **2** and (**b**) the percentages of elemental contacts.

The fingerprint plots (2D representation of a Hirshfeld surface) with percentages for the elements involved in the contacts are presented in Figure 6b. The major interactions are H···H/H···H (44.4%), C···H/H···C (22.3%), N···H/H···N (12.7%), O···H/H···O (11.8%), C···C/C···C (3.6%), C···N/N···C (2.6%), C···O/O···C (1.5%), N···O/O···N (0.9%), and O···O/O···O (0.2%). The contributions from the O···H/H···O, N···H/H···N, and C···H/H···C contacts are represented by a pair of sharp spikes (Figure 6a).

### 2.3. Vibrational Analysis

Infrared (IR) spectroscopy is a robust technique routinely used in the determination of chemical speciation and intermolecular interactions. Spectra from polyatomic compounds can be complex as the numbers of vibrations, for example, depend on factors such as the number of atomic rings and their connectivity. A theoretical and experimental vibrational analysis of compound **2** has therefore been performed. A three-dimensional representation of the molecule, such as coordinates from the crystal structure, is required for the analysis.

**Figure 7:**
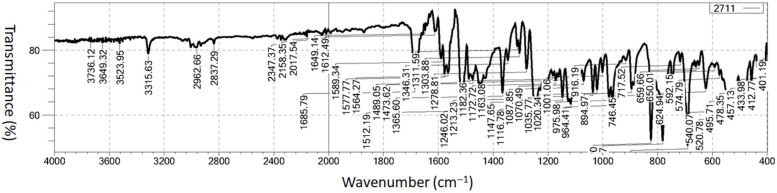
The experimental infrared spectrum of **2**.

For a system with *n* atoms, the number of vibrational modes is 3*n* − 6 [38,39]. Thus compound **2** has 153 vibrational modes as it consists of 53 atoms. The estimated harmonic frequencies were scaled by 0.9614 (DFT-B3LYP) for the 6-311++G(d,p) basis level [40], and all vibrational frequencies were computed with the Gaussian 09W package program [34]. The harmonic modes of compound **2** were calculated in the gaseous phase. The observed and calculated vibrational frequencies in the IR spectrum of compound **2** are given in Table S1. With the help of the VEDA4 tool [41,42], comprehensive potential energy distribution (PED) assignments were acquired. The experimental IR spectrum of **2** is shown in Figure 7. The agreement between the experimental and theoretical results (R^2^) analysis is 0.9079 for the IR data.

#### 2.3.1. Aromatic C–H, Aliphatic (CH_3_) and Aromatic C–C Vibrations

The characteristic C–H modes in heteroaromatic systems fall in the 3100–3000 cm^−1^ range [43]. In the current study, the C–H stretching modes of the aryl rings were computed to be in the 3085–3043 cm^−1^ range using the B3LYP functional and the 6-311++G(d,p) basis set between modes 2 and 14. The PED percentage values of these modes were found to be in the 82–94% range using the VEDA analysis. The C–H stretching modes in the experimental FTIR spectrum were assigned at 3067 cm^−1^.

The asymmetrical and symmetrical stretching vibrations for the aliphatic C–H groups (e.g., CH_3_) were assigned at 3000–2905 cm^−1^ and 2870–2860 cm^−1^, respectively [43,44]. For compound **2**, the aliphatic C–H asymmetric modes were computed at 3023, 3019, 2972, and 2952 cm^−1^ with 84, 99, 93, 99% PED distributions. These modes appeared at 2962 cm^−1^ in the experimental FTIR spectrum. In addition, the symmetrical stretching vibrations of the aliphatic C–H groups (CH_3_) were calculated at 2918 and 2893 cm^−1^ and were observed at 2908 and 2837 cm^−1^ in the experimental FTIR spectrum.

The C–C stretching modes in the aryl rings in compound **2** were assigned as seventeen bands at 1588, 1582, 1570, 1565, 1560, 1549, 1428, 1299, 1297, 1291, 1274, 1230, 1223, 1092, 1067, 1003, and 1000 cm^−1^ using the B3LYP functional and the 6-311++G(d,p) basis set. These bands were observed at 1589, 1577, 1564, 1473, 1303, 1279, 1213, 1088, 1071, and 1001 cm^−1^ in the experimental FTIR spectrum (Table S1). It is evident that the computed aromatic C–C stretching vibrations are in excellent agreement with the experimental results [45].

#### 2.3.2. Carbohydrazide (N–H, N–N, C=N, C=O) and Other C–O Group Vibrations

The N–H stretching vibrations usually appear between 3500 and 3300 cm^−1^ [46]. For *N*′-(4-methoxybenzylidene)-5-phenyl-1*H*-pyrazole-3-carbohydrazide [47], the N5 (hydrazonoic group; N5–H40) stretching vibrations were reported at 3339 cm^−1^. In the current study, the N–H (N46–H47) band in the carbohydrazide group was calculated at 3336 cm^−1^ with 100% contribution B3LYP functional and the 6-311++G(d,p) basis set and was observed at 3316 cm^−1^ in FTIR spectra. The result obtained is in good agreement with the literature [47].

The N–N (N45–N46) stretching modes in the carbohydrazide group were calculated at 1129 and 1107 cm^−1^ with 20 and 10% contributions, respectively, with B3LYP functional and 6-311++G(d,p) basis set. These modes were assigned at 1116 cm^−1^ in the FTIR spectrum which is consistent with those reported [47].

For (*E*)-*N*′-(2,4-dichlorobenzylidene)-5-phenyl-1*H*-pyrazole-3-carbohydrazide [47], the C=N stretching modes associated with the hydrazonoic groups were reported at 1547 cm^−1^. For compound **2**, the νN=C (C22=N45) stretching mode was calculated at 1606 cm^−1^ with 61% PED contribution. The C=N stretching mode was assigned at 1612 cm^−1^ in the FTIR spectrum. Finally, the carbohydrazide group νC=O (C24=O52) stretching mode was computed at 1684 cm^−1^ with 82% PED contribution, and the stretching mode was assigned at 1686 cm^−1^ in the FTIR spectrum. In (*E*)-*N*′-(2,4-dichlorobenzylidene)-5-phenyl-1*H*-pyrazole-3-carbohydrazide [47], νC=O was reported at 1674 cm^−1^ from B3LYP/6-311+G(2d,p). The νC–O (C36–O53), νC–O (C12–O51), νC-O (C1–O51), and νC–O (C41–O53) stretching modes were calculated at 1230, 1223, 1197, and 1015 cm^−1^, respectively, with the B3LYP/6-311++G(d,p) basis set. These modes were assigned at 1213, 1183, and 1020 cm^−1^ in the FTIR spectrum of **2** which is consistent with those reported [47].

#### 2.3.3. 1H-1,2,3-Triazole Ring Vibrations

The N=N, N–N, C–N, and C=N vibrational bands can be difficult to assign precisely since they have a diversity of band structures. The N=N bands were observed at around 1300 cm^−1^ [48], and the N–N bands were reported at around 1000 cm^−1^ [49,50]. In the current work, the νN=N (N48=N49) stretching modes of compound **2** were calculated at 1344, 1328, and 1284 cm^−1^ and were observed in the experimental FTIR spectrum at 1346, 1311, and 1279 cm^−1^, respectively. In addition, the stretching modes of the N–N (N49–N50) bond in the triazole ring were calculated at 1000 and 978 cm^−1^ and were assigned at 1001 and 978 cm^−1^, respectively. The N–C (N50–C26 and N48–C25) stretching vibration modes were calculated at 1487 (N50–C26), 1402 (N50–C26 and N48–C25), 1385 (N50–C26 and N48–C25), 1284 (N48–C25), 1211 (N48–C25), 1183, and 1053 cm^−1^ (N48–C25 and N46–C24). These modes were observed at 1489, 1365, 1279, 1213, 1183, and 1035 cm^−1^ in the FTIR spectrum of **2**. Finally, the νC=C stretching between C25 and C26 was computed at 1538 cm^−1^ with 29% PED contribution and was observed at 1512 cm^−1^ in the FTIR experimental spectrum of **2**.

### 2.4. NMR Chemical Shift Analyses

The experimental ^13^C and ^1^H NMR chemical shifts (DMSO-*d*_6_) of **2** are listed in Table 3. The computational NMR chemical shift results were obtained with the B3LYP/6-311++G(d,p) level using the GIAO method and the IEFPCM solvent model (DMSO) to support and compare with the experimental data. The carbonyl carbons appear very downfield (higher than 155 ppm) in the ^13^C NMR spectra [51,52,53]. The experimental and theoretical chemical shifts for C24 in compound **2** were found at 157.78 ppm and 164.00 ppm, respectively. Imine carbon C22 in the *N*-acylhydrazone group of **2** was detected at 147.68 (exp.)/152.40 (calc.) ppm. The chemical shifts for the carbons in the 1,2,3-triazole ring were at 136.92 (exp.)/145.10 (calc.) for C25 and at 138.53 (exp.)/149.20 (calc.) for C26. The measured and computed NMR chemical shifts for the aryl carbons appear in the 115.98–157.78 ppm region and were computed within the 122.10–168.30 ppm region. Methyl carbon C27 was found at 9.89 (exp.)/12.40 (calc.) ppm, whereas C41 (methoxy carbon) was detected at 55.85 (exp.)/58.60 (calc.) ppm due to the shielding effect of the oxygen atom.

The ^1^H NMR chemical shifts of amide and imino hydrogens for the (*E*)-configuration of the *N*-acylhydrazone derivative were determined at 12.08 ppm and 8.55 ppm with NOESY experimentally [54]. These results are in harmony with the experimental and computed values for the H47 (12.18 (exp.)/10.29 (calc.) ppm) and H23 (8.85 (exp.)/8.21 (calc.) ppm) atoms in compound **2**. The aromatic protons in **2** were experimentally and theoretically found at the regions of 7.10–7.60 ppm and 7.02–8.32 ppm, respectively. The values for H28, H29, and H30 (methyl hydrogens) were 2.11 (exp.)/2.00–3.27 (calc.) ppm, while H42, H43, and H44 (methoxy hydrogens) were found at 3.86 (exp.)/3.82–4.16 (calc.) ppm.

### 2.5. UV–Visible Spectrum and Frontier Orbital Analysis

**Figure 8:**
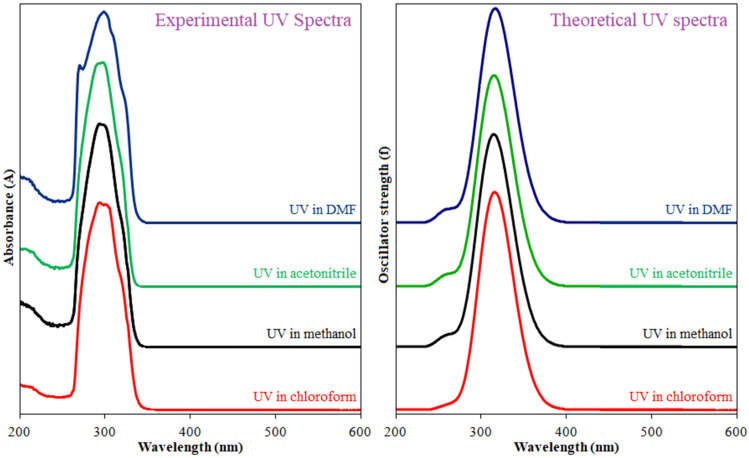
Experimental and simulated UV spectra of **2**.

The UV spectra of compound **2** were measured and simulated in four different solvents, namely chloroform (CHCl_3_), methanol (MeOH), acetonitrile (MeCN), and dimethylformamide (DMF). The experimental and theoretical UV spectra of **2** are shown in Figure 8. The UV spectral parameters (wavelengths, oscillator strengths, excitation energies, and electronic transitions in terms of HOMOs and LUMOs) were computed in the four solvents with the IEFPCM solvent model using the TDDFT/RB3LYP/6-311++G(d,p) computational level of theory. The measured and computed UV spectrum parameters of **2** in the four solvents are listed in Table 4. The percentage contributions computed in terms of HOMOs and LUMOs of electronic transitions corresponding to the computed six UV wavelengths were obtained using the GaussSum 3.0.1 suite [55].

**Figure 9:**
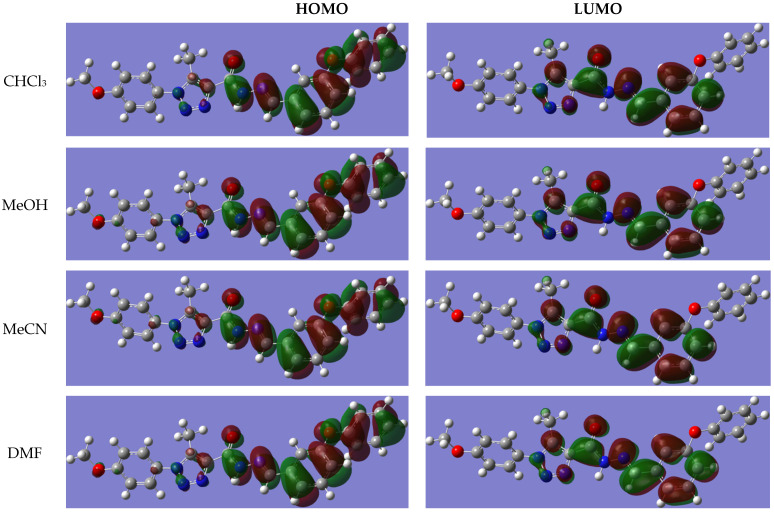
HOMO and LUMO simulations of **2**. Red and green colors indicate negative and positive charges, respectively, within **2**.

Electronic transition from HOMO to LUMO is the lowest energy transition in a molecular system. The wavelength and oscillator strength values for this intramolecular H→L electronic transition in compound **2** were theoretically found at 327.30 nm in CHCl_3_, 327.61 nm in MeOH, 327.75 nm in MeCN, and 328.38 nm in DMF. An increase in solvent polarity leads to a bathochromic shift (red or longer wavelength shift) of the π→π* electronic transition. Since the difference in polarities of the solvents was not very large, the same shift effect was not clearly evident from the experimental UV spectra. Accordingly, the H→L transitions at the low-energy maximum wavelength of compound **2** were calculated in the four solvents corresponding to the π→π* electronic transition. The experimental values corresponding to the computed wavelengths were observed at 293 and 301 nm in CHCl_3_, 293 and 299 nm in MeOH, 292 and 298 nm in MeCN, and 299 nm in DMF. Moreover, the HOMO and LUMO simulations depicted in Figure 9 showed that the HOMO electron localizations are mostly placed over bonding pi electrons (or π) of the aromatic 3-phenoxybenzylidene and 4-carbohydrazide (imino and amide) groups within the compound. Conversely, the LUMO electrons are mainly localized on anti-bonding pi electrons (or π*) of the same molecular groups. The results of the HOMO and LUMO electron localizations simulated in the four solvents of compound **2** confirm the π→π* electronic transition.

LUMO and HOMO can be used to rationalize various molecular properties, such as ionization potential, electron affinity, chemical hardness and softness, excitability, polarizability, acidity, basicity, global reactivity descriptors, electronic and electrical features, electronic transitions, and charge transfers in molecular systems [56,57,58,59,60,61,62]. The computed quantum chemical global molecular descriptors are listed in Table 5. The LUMO and HOMO energy and the |HOMO-LUMO| energy band gap of compound **2** were theoretically obtained as −2.0169, −6.2902, and 4.2733 eV in CHCl_3_, −2.1089, −6.3705, and 4.22616 eV in MeOH, −2.1105, −6.3718, and 4.2613 eV in MeCN, and −2.1111, −6.3724, and 4.2613 eV in DMF, respectively. Clearly, the increase in solvent polarity led to a decrease in these energy values. Similarly, the increase in solvent polarity led to an increase in ionization potential, electron affinity, chemical softness, electronegativity, and electrophilicity index for compound **2**, whereas chemical hardness and potential decreased.

### 2.6. Nonlinear Optical (NLO) Properties

Analysis of NLO is essential in the optimization of materials for some applications [63,64]. The mean polarizability (α_total_), anisotropy of polarizability (Δα), first-order hyperpolarizability (β_0_), and dipole moments of compound **2** were computed using the DFT-B3LYP functional and the 6-311++G(d,p) basis set in different solvents. The urea molecule was chosen as a reference (initial) material. The α_total_, ∆α, and β_0_ values for urea are 5.07643717 × 10^−24^ esu, 2.13568262 × 10^−24^ esu, and 7.2228469891 × 10^−31^ esu, respectively [65]. These parameters were calculated using Equations (1)–(4).

(1) $$\alpha_{\mathrm{total}}=\frac{1}{3}\left(\alpha_{\mathrm{xx}}+\alpha_{\mathrm{yy}}+\alpha_{\mathrm{zz}}\right)$$

(2) $$\Delta \alpha =\frac{1}{\sqrt{2}}{\left[{\left(\alpha_{\mathrm{xx}}-\alpha_{\mathrm{yy}}\right)}^{2}+{\left(\alpha_{\mathrm{yy}}-\alpha_{\mathrm{zz}}\right)}^{2}+{\left(\alpha_{\mathrm{zz}}-\alpha_{\mathrm{xx}}\right)}^{2}+6a_{\mathrm{xz}}^{2}+6a_{\mathrm{xy}}^{2}+6a_{\mathrm{yz}}^{2}\right]}^{1/2}$$

(3) $$\beta_{0}={\left[{\left(\beta_{\mathrm{xxx}}+\beta_{\mathrm{xyy}}+\beta_{\mathrm{xzz}}\right)}^{2}+{\left(\beta_{\mathrm{yyy}}+\beta_{\mathrm{yzz}}+\beta_{\mathrm{yxx}}\right)}^{2}+{\left(\beta_{\mathrm{zzz}}+\beta_{\mathrm{zxx}}+\beta_{\mathrm{zyy}}\right)}^{2}\right]}^{1/2}$$

(4) $$\mu_{\mathrm{total}}={\left(\mu_{x}^{2}+\mu_{y}^{2}+\mu_{z}^{2}\right)}^{1/2}$$

Table 6 shows the NLO values obtained for compound **2**. The order of the dipole moment magnitudes is as follows: CHCl_3_ μ_total_ > MeOH μ_total_ > MeCN μ_total_ > DMF μ_total_. The order of the first-order hyperpolarizabilities (β_0_) is as follows: DMF β_0_ > MeCN β_0_ > MeOH β_0_ > CHCl_3_ β_0_. The mean polarizability (α_total_) was as follows: DMF α_total_ > MeCN α_total_ > MeOH α_total_ > CHCl_3_ α_total_. The anisotropy of polarizability (∆α) was found to be as follows: CHCl_3_ ∆α > MeOH ∆α > MeCN ∆α > DMF ∆α. Additionally, Table 7 lists the α_total_, ∆α, and β_0_ parameters for compound **2** that are many times more powerful than those for the reference substance urea.

### 2.7. Drug-Likeness and ADME Studies

The in silico absorption, distribution, metabolism, and excretion (ADME) analysis used in the current study was performed to determine some physicochemical properties for compound **2**, including lipophilicity, water solubility, pharmacokinetics, drug-likeness, and medicinal chemistry. Taking advantage of the fact that performance of a web-based in silico investigation before experimental analysis can reduce research costs, the assessment was carried out with the aid of web-based SwissADME [66,67]. The bioavailability radar ((i) LIPO (lipophilicity) (−0.7 < XLOGP3 < 5.0), (ii) SIZE (150 g/mol < MW < 500 g/mol), (iii) POLAR (polarity) (20 Å^2^ < TPSA < 130 Å^2^), (iv) INSOLU (insolubility) (0 < logS (ESOL) < 6), (v) INSATU (insaturation) (0.25 < fraction Csp3 < 1), and (vi) FLEX (flexibility) (0 < number of rotatable bonds < 9)) obtained predict the drug-likeness of any molecular system.

**Figure 10:**
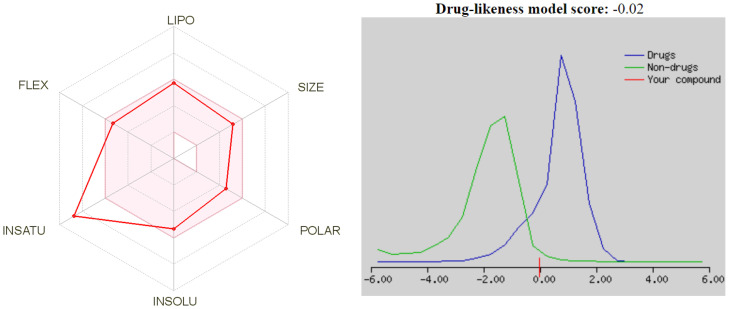
The bioavailability radar (left) [39,66] and drug-likeness model score plots [68] of **2**.

The results obtained for **2** are recorded in Table 8. The LIPO, SIZE, POLAR, INSOLU, INSATU, and FLEX quantities were found to be 4.49, 427.46 g/mol, 90.63 Å^2^, −5.32, 0.08, and 8, respectively. The parameters, except for INSATU, are within the optimal region specified in the bioavailability radar (Figure 10). Compound **2** possibly has an oral drug potential. Similarly, a drug-likeness model score of −0.02 was obtained from the web-based Molsoft application [68]. Assessment using models developed by Lipinski et al. [69], Ghose et al. [70], Veber et al. [71], Egan et al. [72], and Muegge et al. [73], indicated that compound **2** exhibits drug-likeness properties in all models. Lipinski’s rule of five [69] is the simplest and most basic model developed to predict drug-likeness based on the physicochemical properties of molecular systems. According to this model, a suitable molecular system has MW ≤ 500 g/mol, *n*-octanol/water partition coefficient (MlogP) ≤ 5, number of hydrogen bond donors (HBD) ≤ 5, and number of hydrogen bond acceptors (HBA) ≤ 10. In accordance with Lipinski’s rule of five, MW, MlogP, HBD, and HBA values for compound **2** are 427.46 g/mol, 4.14, 1, and 6, respectively. The gastrointestinal (GI) absorption property of **2** is high, whereas the blood–brain barrier (BBB) is permeant and P-glycoprotein (P-gp) substrate activities are not available. The results for the CYP1A2, CYP2D6, CYP2C19, CYP2C9, and CYP3A4 inhibitors are obtained as “no” and “yes”, respectively. The skin permeation (log*K_p_*) of compound **2** has a good value (−5.72 cm/s). These pharmacokinetic properties revealed that compound **2** might have weak-to-moderate biological properties.

### 2.8. Molecular Docking Study

The biological activity assessment of compound **2** was conducted using a web-based PASS online analysis [74,75]. The PASS evaluation works on the basis of the structure activity relationship (SAR) model and provides reliable activity data. The SAR model sets a relationship between the molecular chemical structure and biological activity. According to the PASS analysis, compound **2** has an activity on HMGCS2 (3-hydroxy-3-methylglutaryl-CoA synthase 2 (mitochondrial)) with Pa of 0.775 and Pi of 0.006. The appropriate target macromolecule 2WYA [76] was selected to investigate the activity of **2** on HMGCS2. The high-resolution crystal structure of the target macromolecule 2WYA was taken in the .pdb file format from the RCSB Protein Data Bank website [76,77], while the molecular structure of **2** was from the experimental SCXRD study. The AutoDock Vina software was used to perform the molecular docking analysis [78]. Prior to the analysis, the target macromolecule and **2** were prepared with the Discover Studio Visualizer (DSV) suite [79]. In addition, DSV was used to visualize the intermolecular interactions between the target macromolecule and compound **2**.

The target macromolecule 2WYA contains four chains (A, B, C, and D). The molecular docking process showed that the active sites within the A chain are GLU80, ALA81, GLY82, LYS83, GLY87, GLU132, ALA165, CYS166, TYR200, ASN204, ALA205, THR208, PHE241, GLY255, SER258, TYR262, HIS301, PRO303, PHE304, LYS306, LYS310, ASN380, GLY413, SER414, SER440, and SER443. The molecular docking research space containing these active sites was defined as 50 Å × 58 Å × 30 Å in volume, 0.375 Å as spacing, and 8.2, 49.3, and 19.3 for the x, y, and z centers. The binding affinity and RMSD values calculated for ten different binding poses of **2** docked into the A chain of the target macromolecule 2WYA are given in Table 9.

**Figure 11:**
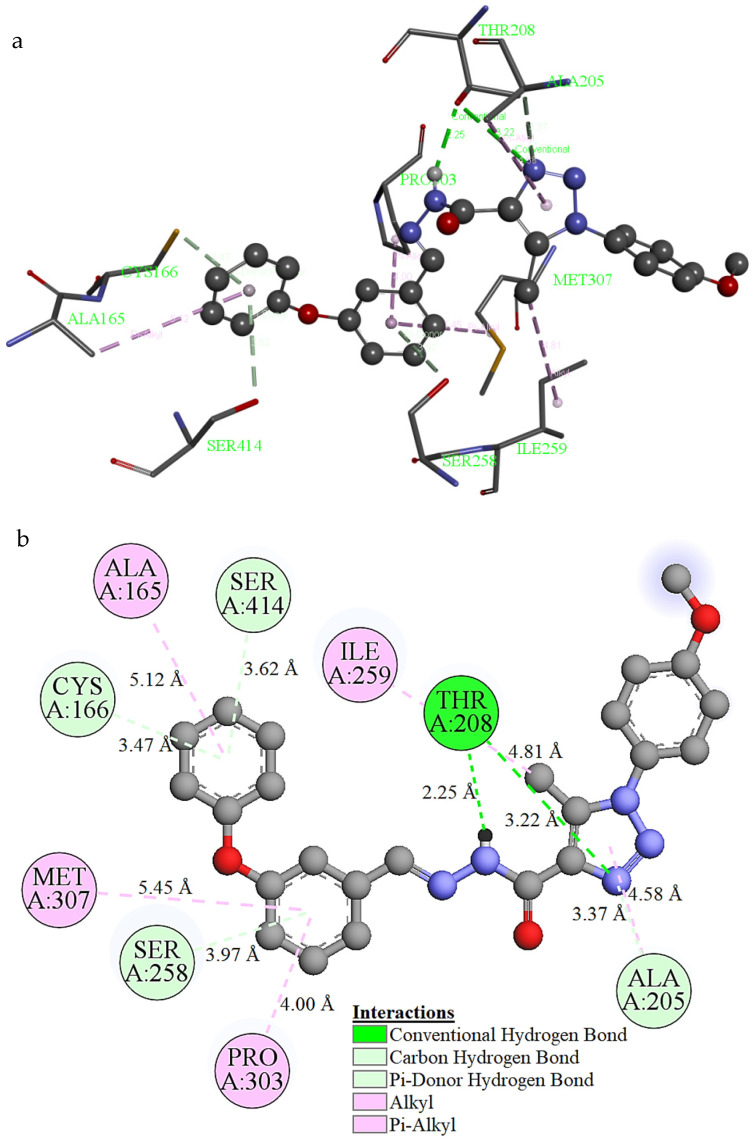
Three-dimensional (**a**) and two-dimensional (**b**) visualizations of intermolecular interactions between **2** and the A chain of the target macromolecule 2WYA.

The binding affinity value of −10.10 kcal/mol for the best conformational pose of **2** indicates a good binding. The 3D and 2D visualizations of the intermolecular interactions are presented in Figure 11, without the hydrogen atoms of both the ligand and the macromolecule. Two conventional hydrogen bond interactions were obtained with the N–H_(ligand)_ ··O_(THR208)_ and O–H_(THR208)_ ··N_(ligand)_ notations with interaction distances of 2.25 Å and 3.22 Å, respectively. One carbon–hydrogen bond was found at the value of 3.37 Å with the C–H_(ALA205)_ ··N_(ligand)_ notation. Three pi-donor hydrogen bond interactions at values of 3.47 Å, 3.62 Å, and 3.97 Å were formed between the aromatic pi-electrons of **2** and the CYS166, SER414, and SER258 residues within the A chain of the target macromolecule, respectively. Four pi-alkyl and one alkyl interactions were found at 4.00 Å, 4.58 Å, 5.12 Å, 5.45 Å, and 4.81 Å values between **2** with the PRO303, ALA205, ALA165, MET307, and ILE259 residues, respectively. As can be seen from Figure 11 and the binding affinity value, compound **2** has good activity on HMGCS2.

## 3. Materials and Methods

### 3.1. Instrumentation

The UV–visible spectrum (190–1100 nm) of **2** in DMSO at 20 °C was performed using a UV-6100 double beam spectrophotometer. The IR spectrum (400–4000 cm^−1^) was recorded on an AIM-9000 Shimadzu spectrometer at 20 °C. The ^1^H (500 MHz) and ^13^C NMR (125 MHz) spectra of compound **2** were recorded on a Bruker AV500 spectrometer in DMSO-*d*_6_ at 20 °C. Single-crystal XRD data were collected on an Agilent SuperNova Dual Atlas diffractometer.

### 3.2. Synthesis of **2**

**Scheme 1:**
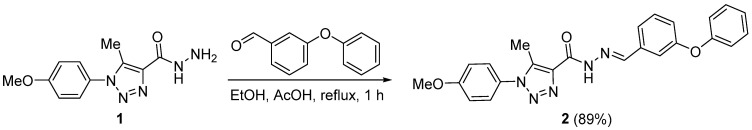
Synthesis of **2**.

A mixture of 1-(4-methoxyphenyl)-5-methyl-1*H*-1,2,3-triazole-4-carbohydrazide (**1**; 0.49 g, 2.0 mmol) and 3-phenoxybenzaldehyde (0.40 g, 2.0 mmol) in anhydrous EtOH (10 mL) containing AcOH (1 mL) was refluxed for 1 h. The mixture was cooled to 20 °C, and the solid obtained was collected by filtration, washed with EtOH (2 × 5 mL), dried, and recrystallized from DMF to give colorless crystals of **2** at 89% yield (Scheme 1), m.p.—256–258 °C. IR (KBr) ν_max_ (cm^−1^): 1589 (C=N), 1612 (C=C), 1649 (C=C), 1686 (C=O), 3316 (NH). ^1^H NMR: δ 2.51 (s, 3H, Me), 3.86 (s, 3H, OMe), 7.10 (d, *J* = 9.2 Hz, 2H, H3/H5 of 4-methoxyphenyl), 7.15 (d, *J* = 7.9 Hz, 1H, H4 of Ar), 7.18 (d, *J* = 8.1 Hz, 2H, H2/H6 of Ph), 7.21 (d, *J* = 8.1 Hz, 1H, H4 of Ph), 7.33 (t, *J* = 7.9 Hz, 1H, H5 of Ar), 7.44 (d, *J* = 8.1 Hz, 2H, H3/H5 of Ph), 7.50 (d, *J* = 7.9 Hz, 1H, H6 of Ar), 7.57 (s, 1H, H2 of Ar), 7.60 (d, *J* = 9.2 Hz, 2H, H2/H6 of 4-methoxyphenyl), 8.58 (s, 1H, CH=N), 12.18 (s, 1H, D_2_O exchange, NH). ^13^C NMR: δ 9.89 (Me), 55.85 (OMe), 115.22 (C3/C5 of 4-methoxyphenyl), 115.98 (C-2 of Ar), 119.47 (C2/C6 of Ph), 120.75 (C4 of Ar), 123.23 (C6 of Ar), 124.32 (C4 of Ph), 127.42 (C2/C6 of 4-methoxyphenyl), 128.46 (C5 of Ar), 130.71 (C3/C5 of Ph), 131.10 (C1 of 4-methoxyphenyl),136.92 (C4 of triazole), 137.44 (C1 of Ar), 138.53 (C5 of triazole), 147.68 (CH=N), 156.76 (C1 of Ph), 157.77 (C-3 of Ar), 157.78 (C=O), 160.68 (C4 of 4-methoxyphenyl). Analysis calculated for C_24_H_21_N_5_O_3_ (427.16): C, 67.44; H, 4.95; N, 16.38. Found: C, 67.56; H, 5.13; N, 16.68%.

### 3.3. Theoretical Details

The DFT approach was used for all quantum chemical computations of compound **2**. Becke’s three-parameter hybrid exchange functional, the Lee–Yang–Parr [80] (B3LYP) functional, and the 6-311++G(d,p) basis set were used within the Gaussian 09 package [34] and GaussView 5.0 programs [35]. The Hirshfeld surface analysis was conducted using the CrystalExplorer tool, which included a visual representation of the d_norm_ map or probable hydrogen bonding, percentage interactions of atoms, and a two-dimensional fingerprint [81]. The AutoDock Vina tool was used to analyze the molecular docking process between the title molecule–ligand and macromolecule 2WYA [78]. The physicochemical properties of compound **2** were evaluated by considering Lipinski rules by online server SwissADME [67].

### 3.4. Crystal Structure Determination

Single-crystal XRD data for **2** were collected at room temperature on an Agilent SuperNova Dual Atlas diffractometer with a mirror monochromator using Cu radiation. The crystal structure was solved using SHELXS [82] and refined using SHELXL [83]. Non-hydrogen atoms were refined with anisotropic displacement parameters. Hydrogen atoms were inserted in idealized positions, and a riding model was used with the U_iso_ set at 1.2 or 1.5 times the value of U_eq_ for the atom to which they are bonded.

The ORTEP-III program [84] was used for the molecular visualization and the PLATON program [85] was used for the identification of hydrogen bonding within the WinGX crystallographic software package [84]. Table 10 shows the refinement data, and the structural details were deposited in the Cambridge Crystallographic Data Centre (CCDC) with under reference number 1031241.

## 4. Conclusions

The optimized geometries and spectral simulations correlate well with the experimental scores. Theoretical and experimental ^13^C and ^1^H chemical shift data were obtained and compared for the molecule. The nonlinear properties of the title molecule were calculated in four different solvents and the resulting α_total_, ∆α, and β_0_ parameters were compared with a reference urea molecule. The drug similarity of the title molecule and the ADME properties were explored. The results indicate that the title molecule has favorable pharmacological properties and a promising therapeutic potential. We believe that this research will contribute to future theoretical and experimental research on similar materials. The molecular docking computations between the ligand and the receptor PDB:2WYA-A chain were conducted with the AutoDock Vina program. The binding affinity value of –10.10 kcal/mol for the best conformational pose of the ligand compound indicates a good binding. As evident from the scores, the ligand/molecule has prospects for good activity on HMGCS2.

## Figures and Tables

**Table 1:** Hydrogen bond geometry (Å, °) of compound **2**.

D–H···A	D–H	H···A	D···A	D–H···A
N2–H2*A*···N3	0.86	2.39	2.763 (5)	107
N7–H7···N8	0.86	2.38	2.752 (5)	107
C17–H17*B*···O2	0.96	2.55	3.105 (7)	117
C41–H41*B*···O5	0.96	2.59	3.133 (7)	116
C13–H13···O2 ^i^	0.93	2.45	3.316 (5)	155
C41–H41*C*···N8^i^	0.96	2.62	3.444 (6)	144
C37–H37···O5 ^ii^	0.93	2.45	3.334 (5)	158
C17–H17*C*···N3 ^ii^	0.96	2.65	3.486 (6)	147

Symmetry codes: (i) x + 1, y, z; (ii) x − 1, y, z.

**Table 2:** Some selected bond distances, bond angles, and torsion angles of **2**.

Structural Parameters	X-ray Diffraction	DFT/B3LYP/6-31++G(d,p)
Bond Distances (Å)		
C1–O1	1.392 (6)	1.38203
C7–O1	1.388 (6)	1.38124
C9–C13	1.441 (6)	1.46417
C13–N1	1.287 (5)	1.28080
N1–N2	1.369 (5)	1.35305
N2–C14	1.363 (5)	1.38069
C14–O2	1.215 (5)	1.21697
C14–C15	1.463 (6)	1.48180
C15–N3	1.363 (5)	1.36541
N3–N4	1.303 (5)	1.29101
N4–N5	1.372 (5)	1.36720
N5–C18	1.427 (6)	1.42794
C21–O3	1.343 (6)	1.35941
O3–C24	1.425 (8)	1.42349
**Bond angles (°)**		
C1–O1–C7	117.3 (4)	120.61383
C9–C13–N1	120.9 (4)	121.94730
C13–N1–N2	115.0 (4)	117.12147
N1–N2–C14	120.8 (4)	121.49785
N2–C14–O2	124.0 (4)	124.86186
N2–C14–C15	113.4 (4)	112.05835
C14–C15–C16	127.9 (4)	128.42862
C16–N5–C18	130.5 (4)	129.54940
N4–N5–C18	118.6 (4)	119.44218
C21–O3–C24	117.2 (5)	118.83652
**Torsion angles (** **°)**		
C1–O1–C7–C12	35.0 (8)	41.52414
O2–C14–C15–C16	14.0 (8)	2.42954
C16–N5–C18–C19	137.2 (5)	128.19985
O3–C21–C22–C23	177.2 (5)	179.59354
O2–C14–N2–N1	1.6 (8)	1.64013

**Table 3:** The experimental and computed ^1^H and ^13^C NMR isotropic chemical shifts of **2** in DMSO-*d*_6_ (related to the TMS as a standard; ppm).

Atom	δ_exp._	δ_calc._	Atom	δ_exp._	δ_calc._
C1	156.76	168.30	H3	7.18	7.45
C2	119.47	127.10	H5	7.44	7.49
C4	130.71	137.00	H7	7.21	7.31
C6	124.32	130.10	H9	7.44	7.51
C8	130.71	136.70	H11	7.18	7.11
C10	119.47	124.50	H14	7.57	8.32
C12	157.77	167.30	H17	7.50	7.38
C13	115.98	122.10	H19	7.33	7.51
C15	137.44	144.80	H21	7.15	7.02
C16	123.23	133.10	H23	8.58	8.21
C18	128.46	137.50	H28	2.11	2.00
C20	120.75	126.30	H29	2.11	3.27
C22	147.68	152.40	H30	2.11	2.37
C24	157.78	164.00	H33	7.60	7.53
C25	136.92	145.10	H35	7.10	7.28
C26	138.53	149.20	H38	7.10	7.07
C27	9.89	12.40	H40	7.60	7.40
C31	131.10	136.20	H42	3.86	3.82
C32	127.42	135.40	H43	3.86	4.16
C34	115.22	125.30	H44	3.86	3.84
C36	160.68	170.00	H47	12.18	10.29
C37	115.22	115.20			
C39	127.42	134.80			
C41	55.85	58.60			

**Table 4:** Experimental absorption wavelengths and computed UV spectral parameters of **2**.

Solvent	λ_exp._ (nm)	Transitions	λ_calc._ (nm)	f	ΔE (eV)	Computed Major Transitions
CHCl_3_	293 and 301	π→π*	327.30	0.6290	3.7881	H→L (93%)
		π→π*	308.20	0.7463	4.0228	H-1→L (90%)
		π→π*	287.25	0.0088	4.3163	H-2→L (94%)
		π→π*	271.87	0.0001	4.5604	H-6→L (44%), H-7→L (35%)
		π→π*	271.00	0.0078	4.5750	H-3→L (72%)
		π→π*	263.06	0.0236	4.7132	H→L + 1 (83%), H-1→L + 1 (12%)
MeOH	293 and 299	π→π*	327.61	0.5721	3.7845	H→L (92%)
		π→π*	308.08	0.7446	4.0244	H-1→L (69%), H-2→L (24%)
		π→π*	293.23	0.0560	4.2283	H-2→L (71%), H-1→L (27%)
		π→π*	271.83	0.0079	4.5612	H-3→L (71%), H-4→L (11%)
		π→π*	269.00	0.0003	4.6091	H-7→L (73%), H-6→L (13%)
		π→π*	259.93	0.0572	4.7699	H→L + 1 (68%), H-1→L + 1 (22%)
MeCN	292 and 298	π→π*	327.75	0.5797	3.7829	H→L (93%)
		π→π*	308.21	0.7388	4.0227	H-1→L (68%), H-2→L (25%)
		π→π*	293.35	0.0552	4.2264	H-2→L (71%), H-1→L (28%)
		π→π*	271.85	0.0080	4.5607	H-3→L (71%), H-4→L (11%)
		π→π*	268.96	0.0003	4.6098	H-7→L (73%), H-6→L (13%)
		π→π*	259.91	0.0594	4.7703	H→L + 1 (67%), H-1→L + 1 (23%)
DMF	299	π→π*	328.38	0.6195	3.7757	H→L (94%)
		π→π*	308.81	0.7133	4.0149	H-1→L (67%), H-2→L (27%)
		π→π*	293.55	0.0459	4.2236	H-2→L (69%), H-1→L (29%)
		π→π*	271.90	0.0083	4.5599	H-3→L (71%), H-4→L (11%)
		π→π*	268.95	0.0003	4.6099	H-7→L (73%), H-6→L (13%)
		π→π*	259.98	0.0656	4.7690	H→L + 1 (66%), H-1→L + 1 (24%)

λ; wavelength, A; absorbance, f; oscillator strength, ΔE; excitation energy.

**Table 5:** Some quantum chemical molecular descriptors computed for **2** in different solvents.

Parameters	CHCl_3_ (ε = 4.7113)	MeOH (ε = 32.613)	MeCN (ε = 35.688)	DMF (ε = 37.219)
*E*_LUMO_ (eV)	−2.0169	−2.1089	−2.1105	−2.1111
*E*_HOMO_ (eV)	−6.2902	−6.3705	−6.3718	−6.3724
Energy bandgap |*E*_HOMO_−*E*_LUMO_| (eV)	4.2733	4.2616	4.2613	4.2613
Ionization potential (*I* = −*E*_HOMO_) (eV)	6.2902	6.3705	6.3718	6.3724
Electron affinity (*A* = −*E*_LUMO_) (eV)	2.0169	2.1089	2.1105	2.1111
Chemical hardness (*η* = (*I* − *A*)/2) (eV)	2.1366	2.1308	2.1307	2.1307
Chemical softness (*ζ* = 1/2*η*) (eV^−1^)	0.2340	0.2347	0.2347	0.2347
Electronegativity (*χ* = (*I* + *A*)/2) (eV)	4.1535	4.2397	4.2412	4.2417
Chemical potential (*μ* = −(*I* + *A*)/2) (eV)	−4.1535	−4.2397	−4.2412	−4.2417
Electrophilicity index (*w* = *μ*^2^/2*η*) (eV)	4.0372	4.2179	4.2211	4.2222
Maximum charge transfer index (Δ*N_max._* = −*μ*/*η*)	1.9440	1.9897	1.9905	1.9908

**Table 6:** The electric dipole moment, polarizability, and first-order hyperpolarizability values of **2** in different solvents.

MeCN				CHCl_3_			
P	Value (esu)	P	Value (esu)	P	Value (esu)	P	Value (esu)
α_xx_	1.06054264 × 10^−22^	β_xxx_	2.7232392281 × 10^−29^	α_xx_	1.02029387 × 10^−22^	β_xxx_	2.3952996201 × 10^−29^
α_xy_	3.34406970 × 10^−24^	β_xyy_	−6.7163351322 × 10^−30^	α_xy_	2.71384369 × 10^−24^	β_xyy_	−5.3041591257 × 10^−30^
α_xz_	−1.96406718 × 10^−25^	β_xzz_	3.9775500915 × 10^−31^	α_xz_	−1.74450518 × 10^−26^	β_xzz_	3.4264850753 × 10^−31^
α_yy_	6.14781650 × 10^−23^	β_yyy_	2.5742850132 × 10^−30^	α_yy_	5.53813340 × 10^−23^	β_yyy_	1.9066353494 × 10^−30^
α_yz_	5.61089698 × 10^−24^	β_yxx_	−4.2660804480 × 10^−30^	α_yz_	4.95659385 × 10^−24^	β_yxx_	−3.8201464576 × 10^−30^
α_zz_	4.89191925 × 10^−23^	β_yzz_	4.1525419899 × 10^−31^	α_zz_	4.34111794 × 10^−23^	β_yzz_	2.7472139962 × 10^−31^
α_total_	72.1505403 × 10^−24^	β_zzz_	−7.2495596707 × 10^−31^	α_total_	66.9406335 × 10^−24^	β_zzz_	−4.4045838309 × 10^−31^
Δα	53.2230869 × 10^−24^	β_zxx_	−6.2103327791 × 10^−31^	Δα	54.5298916 × 10^−23^	β_zxx_	−4.5456621571 × 10^−31^
μ_x_	−1.9477408	β_zyy_	4.0011609701 × 10^−31^	μ_x_	−1.9675829	β_zyy_	2.6810099604 × 10^−31^
μ_y_	0.2212355	β_0_	209.74073818 × 10^−31^	μ_y_	0.2212355	β_0_	190.72367172 × 10^−31^
μ_z_	0.079071			μ_z_	0.0277634		
μ_total_	1.9618592			μ_total_	1.9801764		
**MeOH**				**DMF**			
**P**	**Value (esu)**	**P**	**Value (esu)**	**P**	**Value (esu)**	**P**	**Value (esu)**
α_xx_	1.05991420 × 10^−22^	β_xxx_	2.7186313156 × 10^−29^	α_xx_	1.06081731 × 10^−22^	β_xxx_	2.7252481965 × 10^−29^
α_xy_	3.33205805 × 10^−24^	β_xyy_	−6.6900564567 × 10^−30^	α_xy_	3.34934748 × 10^−24^	β_xyy_	−6.7278538996 × 10^−30^
α_xz_	−1.93663892 × 10^−25^	β_xzz_	3.9658089890 × 10^−31^	α_xz_	−1.97595431 × 10^−25^	β_xzz_	3.9828977750 × 10^−31^
α_yy_	6.13687028 × 10^−23^	β_yyy_	2.5625263928 × 10^−30^	α_yy_	6.15261523 × 10^−23^	β_yyy_	2.5794420947 × 10^−30^
α_yz_	5.60026327 × 10^−24^	β_yxx_	−4.2586482569 × 10^−30^	α_yz_	5.61554695 × 10^−24^	β_yxx_	−4.2692974780 × 10^−30^
α_zz_	4.88130109 × 10^−23^	β_yzz_	4.1222501073 × 10^−31^	α_zz_	4.89658696 × 10^−23^	β_yzz_	4.1658927840 × 10^−31^
α_total_	72.0577113 × 10^−24^	β_zzz_	−7.1870785944 × 10^−31^	α_total_	72.1912509 × 10^−24^	β_zzz_	−7.2771077289 × 10^−31^
Δα	53.2598403 × 10^−24^	β_zxx_	−6.1728143986 × 10^−31^	Δα	53.2068187 × 10^−24^	β_zxx_	−6.2270660838 × 10^−31^
μ_x_	−1.9482024	β_zyy_	3.9736307175 × 10^−31^	μ_x_	−1.9475465	β_zyy_	4.0132371478 × 10^−31^
μ_y_	0.2190976	β_0_	209.53283113 × 10^−31^	μ_y_	0.2221764	β_0_	209.83099781 × 10^−31^
μ_z_	0.0777472			μ_z_	0.0796567		
μ_total_	1.9620247			μ_total_	1.9617963		

P, parameters.

**Table 7:** The α_total_, ∆α, and β_0_ parameters for compound **2** as a multiple of the values for urea (i.e., value **2**/value (urea)).

Value	MeCN	CHCl_3_	MeOH	DMF
α_total_	14.21	13.19	14.19	14.22
∆α	24.92	25.53	24.94	24.91
β_0_	29.04	26.41	29.01	29.05

**Table 8:** Physicochemical, lipophilicity, water solubility, pharmacokinetic, drug-likeness, and medicinal chemistry properties obtained from the SwissADME website for **2**.

SMILES	COc1ccc(cc1)n1nnc(c1C)C(=O)N/N=C/c1cccc(c1)Oc1ccccc1
Physicochemical Properties	Values
Molecular weight (MW)	427.16 g/mol
Number of heavy atoms	32
Number of aromatic heavy atoms	23
Fraction Csp^3^	0.08
Number of rotatable bonds	8
Number of H-bond acceptors (HBA)	6
Number of H-bond donors (HBD)	1
Molar refractivity (MR)	120.50
Topological polar surface area (TPSA)	90.63 Å^2^
**Lipophilicity**	**Values**
Log*P*_o/w_ (iLOGP)	3.82
Log*P*_o/w_ (XLOGP3)	4.49
Log*P*_o/w_ (WLOGP)	4.14
Log*P*_o/w_ (MLOGP)	3.00
Log*P*_o/w_ (SILICOS-IT)	3.59
Consensus Log*P*_o/w_	3.81
**Water solubility**	**Values**
Log *S* (ESOL)	−5.32
Solubility	2.03 × 10^−3^ mg/mL; 4.76 × 10^−6^ mol/L
Class	Moderately soluble
Log*S* (Ali)	−6.11
Solubility	3.29 × 10^−4^ mg/mL; 7.70 × 10^−7^ mol/L
Class	Poorly soluble
Log*S* (SILICOS-IT)	−7.83
Solubility	6.25 × 10^−6^ mg/mL; 1.46 × 10^−8^ mol/L
Class	Poorly soluble
**Pharmacokinetics**	**Values**
Gastrointestinal (GI) absorption	High
Blood–brain barrier (BBB) permeant	No
P-glycoprotein (P-gp) substrate	No
CYP1A2 inhibitor	No
CYP2C19 inhibitor	Yes
CYP2C9 inhibitor	Yes
CYP2D6 inhibitor	No
CYP3A4 inhibitor	Yes
Log*K*_p_ (skin permeation)	−5.72 cm/s
**Drug-likeness**	**Values**
Lipinski	Yes; zero violation
Ghose	Yes
Veber	Yes
Egan	Yes
Muegge	Yes
Bioavailability score	0.55
**Medical chemistry**	**Values**
PAINS	0 alert
Brenk	1 alert: imine_1
Lead-likeness	No; three violations: MW > 350, rotors > 7; XLOGP3 > 3.5
Synthetic accessibility	3.52

**Table 9:** The AutoDock Vina results for ten different poses of **2** docked into the A chain of the target macromolecule 2WYA.

Mode	Binding Affinity (kcal/mol)	Distance from the Best Mode (Å)	
		RMSD/lb	RMSD/ub
1	−10.10	0.000	0.000
2	−9.90	2.271	9.987
3	−9.80	1.851	10.125
4	−9.60	2.361	10.036
5	−9.50	3.371	9.958
6	−9.10	1.826	10.625
7	−9.00	14.962	17.748
8	−8.80	2.062	2.664
9	−8.70	2.603	4.106
10	−8.60	15.096	18.245

**Table 10:** Crystallographic data of **2**.

Chemical Formula	C_24_H_21_N_5_O_3_
Formula weight	426.46
F(000)	896
Radiation	CuK_α_ (*λ* = 1.54184 Å)
Crystal system	Triclinic
Space group	*P* $\bar{1}$
*a*, *b*, *c* (Å)	6.6452 (5), 7.4466 (5), 43.403 (2)
α, β, γ (°)	86.250 (4), 89.193 (5), 80.484 (6)
Volume (Å^3^)	2113.7 (2)
*Z*	4
Crystal dimension (mm)	0.30 × 0.18 × 0.03
Temperature (*K*)	293
*D_calc_* (mg m^−3^)	1.343
Measured reflections	9077
Independent reflections	9077
Number of reflections with I > 2 *σ(I)*	6949
*θ* range (°)	6.0–77.1
Range of h, k, l	−8 < h < 8, −9 < k < 9, −54 < l < 52
Refinement on	*F^2^*
Calculated weights	*W* = 1/(σ^2^(*Fo*^2^) + (0.1235P)^2^ + 1.0892P), where P = (*Fo*^2^ + 2*Fc*^2^/3)
Number of refinement parameters	582
*S*	1.08
*R* (*F*^2^ > 2*σ*(*F*^2^))	0.075
*wR* (*F*^2^)	0.241
(Δρ)_max_ (e Å^−3^)	0.30
(Δρ)_min_ (e Å^−3^)	−0.23

## Data Availability

Data are contained within the article.

## Supplementary Materials

The following supporting information can be downloaded at https://www.mdpi.com/article/10.3390/molecules27072193/s1. Table S1: observed and calculated IR vibrational frequencies of **2**; procedure to calculate the HOMO and LUMO, energy gap, and global reactivity; parameters; CIF of **2**; UV spectral data of **2** in different solvents; IR spectra of **2**; NMR spectra of **2**.

## Sample Availability

A sample of compound **2** is available from the authors.
