# Supplementary material for: Quantum Computational Investigation of (E)-1-(4-methoxyphenyl)-5-methyl-N′-(3-phenoxybenzylidene)-1H-1,2,3-triazole-4-carbohydrazide
Source: Molecules. 2022 Mar 28;27(7):2193. doi: 10.3390/molecules27072193 (PMC9000758; doi:10.3390/molecules27072193)
Supplement: Supplementary file 1 [file molecules-27-02193-s001.zip › IR peaks.pdf]

SHIMADZU

VME6 03/08/2021 10:45:07

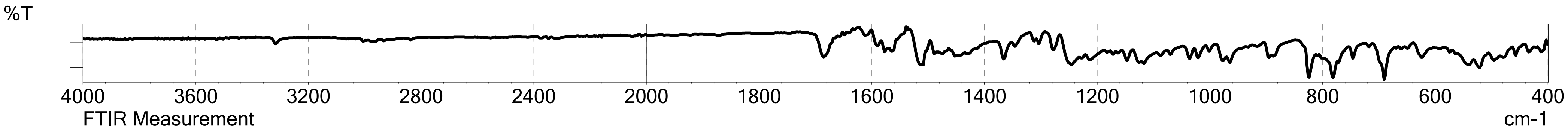

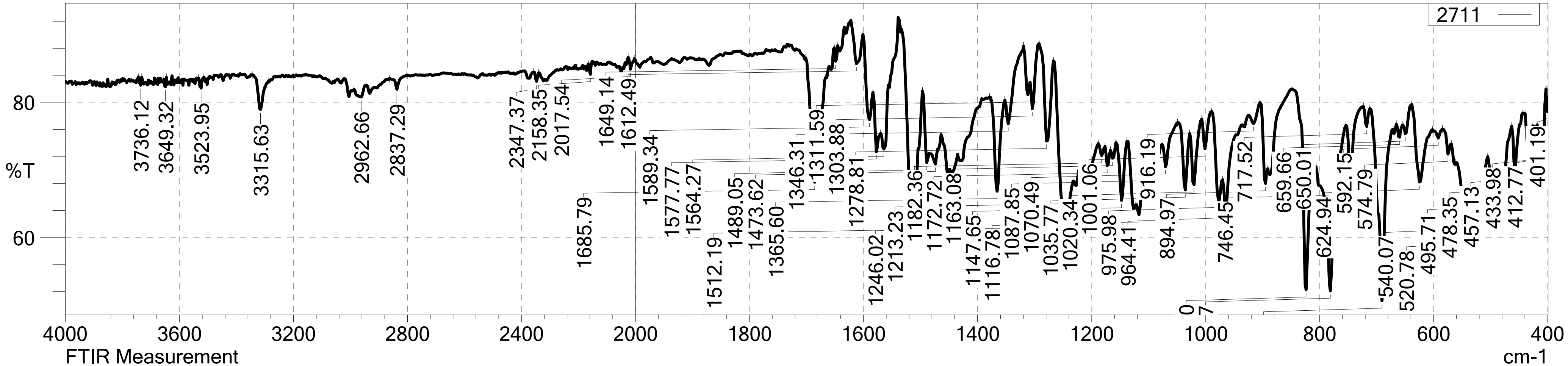

	Item	Value
	Acquired Date&Time	14/07/2021 11:05:33
	Acquired by	System Administrator
	Filename	C:\Users\Shimadzu\Desktop\BK\140721\2711.ispd
	Spectrum name	2711
	Sample name	gb
	Sample ID	271
	Option	
	Comment	FTIR Measurement
	No. of Scans	20
	Resolution	4 [cm-1]
	Apodization	Happ-Genzel

No.	Peak	Intensity	Corr. Intensity	Base (H)	Base (L)	Area	Corr. Area	Comment
1	401.19	78.66	2.72	403.12	399.26	77.078	5.247	
2	412.77	72.64	6.03	418.55	403.12	381.840	63.042	
3	433.98	72.52	5.06	439.77	426.27	343.730	39.233	
4	457.13	69.75	7.34	462.92	443.63	487.093	52.537	
5	478.35	68.20	3.58	486.06	462.92	659.510	33.750	
6	495.71	65.72	4.97	505.35	486.06	615.834	50.534	
7	520.78	60.25	8.53	532.35	505.35	929.010	96.762	
8	540.07	62.34	5.30	561.29	532.35	982.435	78.024	
9	574.79	72.35	2.34	584.43	569.00	396.202	9.914	
10	592.15	74.68	1.34	601.79	584.43	425.203	8.816	
11	624.94	68.20	10.11	638.44	601.79	974.361	161.398	
12	650.01	75.36	2.07	653.87	638.44	349.000	12.415	

13	659.66	74.85	1.89	665.44	653.87	279.436	10.239	
14	690.52	50.63	26.85	711.73	671.23	1282.166	372.970	
15	717.52	76.43	2.81	723.31	711.73	255.504	15.258	
16	746.45	67.44	9.87	754.17	723.31	751.605	72.987	
17	781.17	52.09	14.35	806.25	773.46	1204.233	149.420	
18	823.60	52.28	21.92	848.68	812.03	1051.406	182.678	
19	894.97	68.00	4.98	904.61	891.11	363.463	26.921	
20	916.19	76.85	1.97	923.90	904.61	424.997	20.172	
21	964.41	64.01	6.19	970.19	947.05	690.009	38.695	
22	975.98	65.53	6.05	989.48	970.19	566.327	57.231	
23	1001.06	73.12	4.79	1006.84	989.48	413.628	33.418	
24	1020.34	67.80	8.36	1028.06	1006.84	571.821	72.333	
25	1035.77	67.03	8.95	1049.28	1028.06	576.553	71.631	
26	1070.49	70.46	4.03	1076.28	1049.28	695.603	31.576	
27	1087.85	69.52	3.19	1095.57	1076.28	548.401	25.538	
28	1116.78	63.36	3.03	1122.57	1095.57	867.604	15.068	
29	1147.65	65.47	8.66	1157.29	1138.00	570.601	71.762	
30	1163.08	71.72	1.65	1166.93	1157.29	264.676	8.955	
31	1172.72	70.65	2.60	1176.58	1166.93	271.589	13.087	
32	1182.36	72.12	1.57	1188.15	1176.58	313.219	8.764	
33	1213.23	66.12	5.14	1220.94	1188.15	991.488	79.213	
34	1246.02	62.59	12.69	1267.23	1232.51	1045.285	255.608	
35	1278.81	74.28	13.07	1292.31	1267.23	461.621	146.755	
36	1303.88	79.05	5.38	1307.74	1292.31	248.873	30.712	
37	1311.59	81.12	3.66	1319.31	1307.74	188.722	22.962	
38	1346.31	76.83	4.91	1352.10	1319.31	557.643	44.073	
39	1365.60	66.84	13.70	1379.10	1352.10	670.755	145.263	
40	1473.62	70.87	2.78	1481.33	1463.97	477.613	22.944	
41	1489.05	71.09	4.41	1496.76	1485.19	287.516	21.827	
42	1512.19	62.25	23.26	1533.41	1496.76	968.735	464.477	
43	1564.27	73.10	5.35	1570.06	1556.55	330.846	46.795	
44	1577.77	72.68	6.21	1583.56	1570.06	326.396	35.479	
45	1589.34	77.41	6.93	1600.92	1583.56	319.748	72.766	
46	1612.49	85.71	5.49	1622.13	1600.92	253.818	65.076	
47	1649.14	86.10	2.08	1651.07	1645.28	75.726	7.591	

48	1685.79	68.18	17.11	1701.22	1666.50	819.540	303.408	
49	2017.54	84.90	1.69	2025.26	2004.04	300.110	15.012	
50	2158.35	84.15	1.76	2166.06	2152.56	200.648	10.123	
51	2347.37	83.11	1.31	2357.01	2339.65	280.203	9.910	
52	2837.29	81.92	1.80	2862.36	2802.57	997.203	26.274	
53	2962.66	80.81	1.45	2987.74	2945.30	789.224	34.225	
54	3315.63	78.95	4.71	3342.64	3286.70	1011.433	96.394	
55	3523.95	82.11	1.55	3533.59	3514.30	327.982	12.649	
56	3649.32	82.31	1.30	3655.11	3645.46	164.255	5.868	
57	3736.12	82.54	1.20	3743.83	3732.26	195.563	7.543	
