# Supplementary material for: Quantum Computational Investigation of (E)-1-(4-methoxyphenyl)-5-methyl-N′-(3-phenoxybenzylidene)-1H-1,2,3-triazole-4-carbohydrazide
Source: Molecules. 2022 Mar 28;27(7):2193. doi: 10.3390/molecules27072193 (PMC9000758; doi:10.3390/molecules27072193)
Supplement: Supplementary file 1 [file molecules-27-02193-s001.zip › IR Spectra.pdf]

SHIMADZU

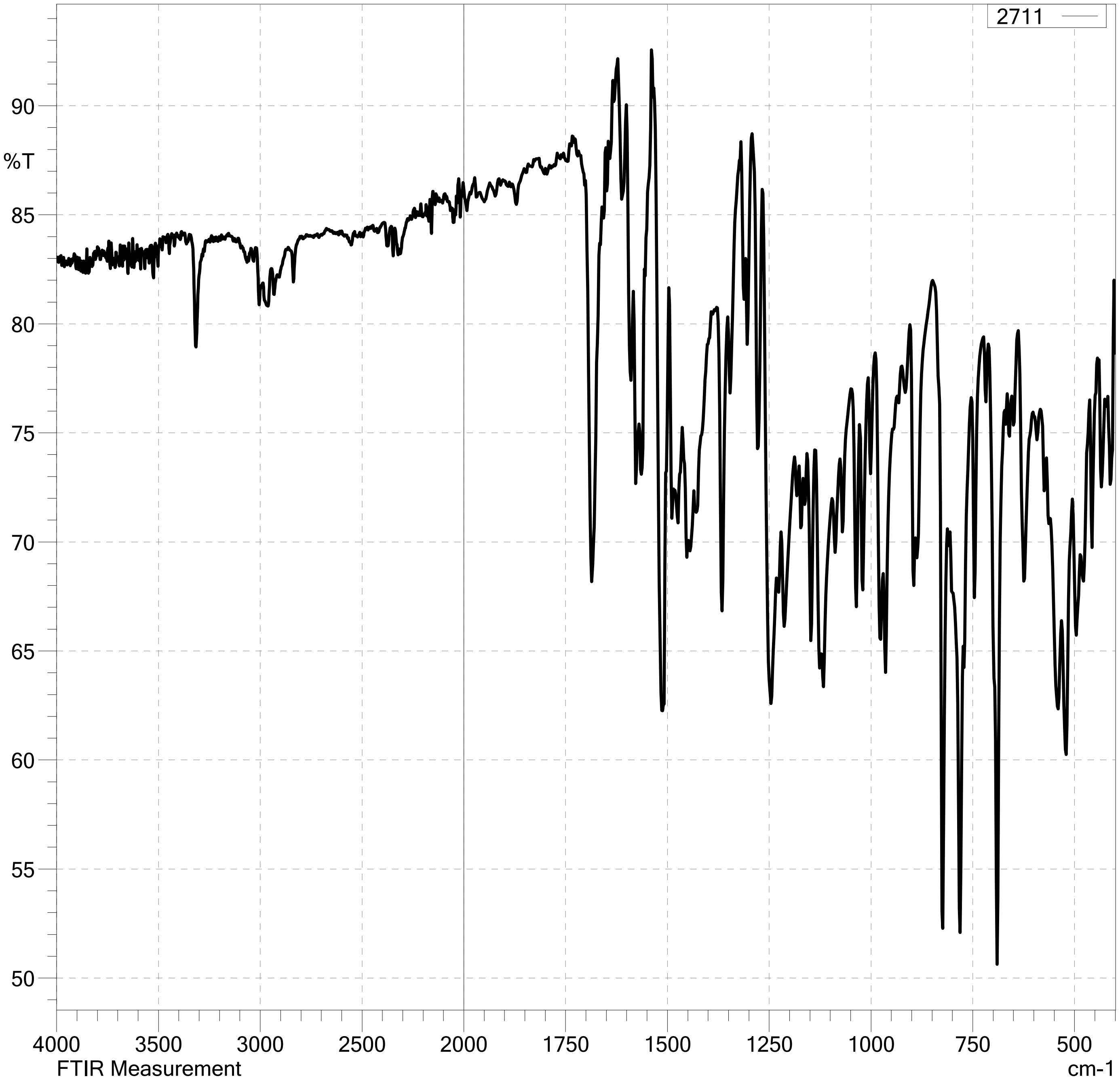

C:¥Users¥Shimadzu¥Desktop¥BK¥140721¥2711.ispd

FTIR Measurement

	Item	Value
2	Sample name	gb
3	Sample ID	271
4	Option	
5	Intensity Mode	%Transmittance
6	Apodization	Happ-Genzel
9	No. of Scans	20

10	Resolution	4 cm-1
