# Supplementary material for: Quantum Computational Investigation of (E)-1-(4-methoxyphenyl)-5-methyl-N′-(3-phenoxybenzylidene)-1H-1,2,3-triazole-4-carbohydrazide
Source: Molecules. 2022 Mar 28;27(7):2193. doi: 10.3390/molecules27072193 (PMC9000758; doi:10.3390/molecules27072193)
Supplement: Supplementary file 1 [file molecules-27-02193-s001.zip › Procedure to Calculate the HOMO and LUMO.pdf]

Procedure to Calculate the HOMO and LUMO, Energy gap, and Global Reactivity Parameters

The molecular orbitals and their properties such as energy are useful for physicists and chemists. This is also used in frontier electron density for predicting the most reactive position in π-electron systems and also explains several types of reactions in conjugated systems. The FMO analysis is widely employed to explain the optical and electronic properties of organic compounds. Knowledge of the HOMO and LUMO, and their properties namely their energy, is very useful to gauge the chemical reactivity of molecules. During molecular interactions, the LUMO accepts electrons and its energy corresponds to the electron affinity (EA), while the HOMO represents electron donors and its energy is associated with the ionization potential (IP). The HOMO-LUMO energy gap explains the concluding charge transfer interaction within the molecule and is useful in determining molecular electrical transport properties. A molecule with a high frontier orbital gap (HOMO-LUMO energy gap) has low chemical reactivity and high kinetic stability because it is energetically unfavorable to add an electron to the high-lying LUMO in order to remove electrons from the low-lying HOMO. For instance, compounds that have a high HOMO-LUMO energy gap are stable, and hence are chemically harder than compounds having a small HOMO-LUMO energy gap.

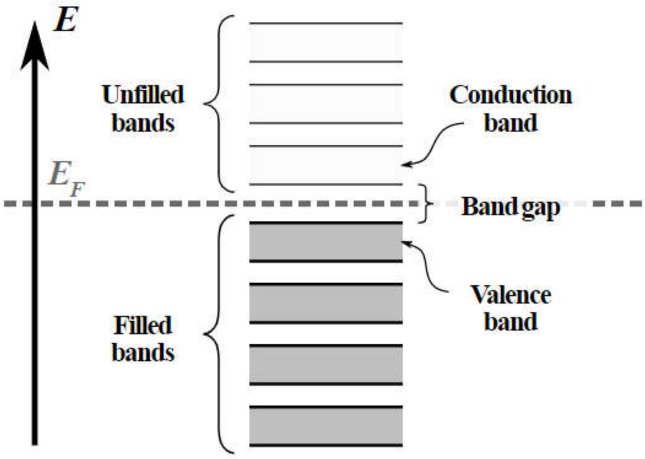

E_{HOMO} and E_{LUMO} are employed in the DFT theorem to estimate the ionization potential (I) and electron affinity (A) given by Koopman's theorem and $E=|E_{HOMO}- E_{LUMO}|$ is utilized to determine the energy bandgap.

$$I = -E_{HOMO} \quad (1)$$
$$A = -E_{LUMO} \quad (2)$$

Using the highest occupied molecular orbital (EHOMO) and lowest unoccupied molecular orbital (ELUMO) energies, the chemical potential (μ) and hardness (⊚) values can be computed using the Koopmans theorem.

$$\text{Chemical Potential} = \mu = -\left(\frac{I+A}{2}\right) = \left(\frac{E_{HOMO}+E_{LUMO}}{2}\right) \quad (3)$$
$$\text{Chemical Hardness} = \eta = \left(\frac{I-A}{2}\right) = \left(\frac{E_{HOMO}-E_{LUMO}}{2}\right) \quad (4)$$

The electrophilicity index (ω) is a measurement of the energy loss produced by the maximal electron flow between acceptor and donor. It is computed as follows:

$$\text{Electrophilicity index } = \omega = \frac{\mu^2}{2\eta} \quad (5)$$

As seen in Equation (6), the negative of the chemical potential is expressed as electronegativity (χ):

$$\text{Electronegativity} = \chi = \left(\frac{I+A}{2}\right) = -\left(\frac{E_{HOMO}+E_{LUMO}}{2}\right) \quad (6)$$

Chemical softness is the inverse of hardness and is calculated as:

$$Chemical\ softness = \xi = \frac{1}{2\eta} = \frac{1}{(\mathrm{E_{HOMO}} - \mathrm{E_{LUMO}})} \quad (7)$$
