# Supplementary material for: Quantum Computational Investigation of (E)-1-(4-methoxyphenyl)-5-methyl-N′-(3-phenoxybenzylidene)-1H-1,2,3-triazole-4-carbohydrazide
Source: Molecules. 2022 Mar 28;27(7):2193. doi: 10.3390/molecules27072193 (PMC9000758; doi:10.3390/molecules27072193)
Supplement: Supplementary file 1 [file molecules-27-02193-s001.zip › Table S1.pdf]

molecules

MDPI

Article

Quantum Computational Investigation of (*E*)-1-(4-Methoxyphenyl)-5-methyl-*N′*-(3-phenoxybenzylidene)-1*H*-1,2,3-triazole-4-carbohydrazide

Halil Gökce [1,*], Fatih Şen [2], Yusuf Sert [2,3], Bakr F. Abdel-Wahab [4], Benson M. Kariuki [5] and Gamal A. El-Hiti [6,*]

1 Vocational School of Health Services, Giresun University, 28200, Giresun, Turkey
2 Sorgun Vocational School, Bozok University, 66700, Yozgat, Turkey; fatihsen55@gmail.com
3 Department of Physics, Faculty of Art & Science, Bozok University, 66900, Yozgat, Turkey; yusufsert1984@gmail.com
4 Applied Organic Chemistry Department, National Research Centre, Dokki, Giza 12622, Egypt; bakrfatehy@yahoo.com
5 School of Chemistry, Cardiff University, Main Building, Park Place, Cardiff, CF10 3AT, UK; kariukib@cardiff.ac.uk
6 Department of Optometry, College of Applied Medical Sciences, King Saud University, Riyadh 11433, Saudi Arabia
* Correspondence: Correspondence: halil.gokce@giresun.edu.tr (H.G.); gelhiti@ksu.edu.sa (G.A.E.-H.); Tel.: +966-11469-3778 (G.A.E.-H.); Fax: +966-11469-3536 (G.A.E.-H.)

Table S3. Observed and calculated IR vibrational frequencies of **2**.

Vib. no.	FTIR	B3LYP	Assignment [a]	Intensity
ν_1	3316	3336	νNH(100) in the N46—H47 carbohydrazide group	27.83
ν_2		3091	νCH(92) in the aromatic ring	4.55
ν_3		3085	νCH(94) in the aromatic ring	0.49
ν_4		3084	νCH(83) in the aromatic ring	3.76
ν_5		3076	νCH(92) in the aromatic ring	3.74
ν_6		3074	νCH(90) in the aromatic ring	1.73
ν_7		3072	νCH(91) in the aromatic ring	7.83
ν_8	3067	3071	νCH(82) in the aromatic ring	0.17
ν_9		3069	νCH(92) in the aromatic ring	9.74
ν_{10}		3064	νCH(83) in the aromatic ring	25.74
ν_{11}		3058	νCH(92) in the aromatic ring	18.91
ν_{12}		3051	νCH(88) in the aromatic ring	9.77
ν_{13}		3043	νCH(94) in the aromatic ring	2.27
ν_{14}		3043	νCH(90) in the aromatic ring	4.81
ν_{15}		3023	νCH_3(84) in the aliphatic group asym. mode	0.08
ν_{16}	2962	3019	νCH_3(99) in the aliphatic group asym. mode	20.76
ν_{17}		2972	νCH_3(93) in the aliphatic group asym. mode	8.01
ν_{18}		2952	νCH_3(99) in the aliphatic group asym. mode	32.87
ν_{19}	2908	2918	νCH_3(99) in the aliphatic group sym. mode	16.64
ν_{20}	2837	2907	νCH(100) in the C22—H23 carbohydrazide group	53.21
ν_{21}	2837	2893	νCH_3(99) in the aliphatic group sym. mode	57.31
ν_{22}	1686	1684	νOC(82) in the C24=O52 carbohydrazide group	296.72
ν_{23}	1612	1606	νNC(61) in the N45=C22 carbohydrazide group	2.02
ν_{24}		1588	νCC(52) in the aromatic ring	49.05
ν_{25}		1582	νCC(22) in the aromatic ring	3.25
ν_{26}	1589, 1577, 1564,	1570	νCC(41) in the aromatic ring	57.63
ν_{27}		1565	νCC(47) in the aromatic ring + δCCC(22)	11.47
ν_{28}		1560	νCC(56) in the aromatic ring	61.81
ν_{29}		1549	νCC(36) in the aromatic ring	104.08
ν_{30}	1512	1538	δCNN(35) + νCC(29) in the C25=C26	293.57
ν_{31}		1501	δHNN(52)	366.64
ν_{32}	1489	1487	δHCC(46) + δCCC(13) + νNC(10) in the triazole ring	446.87
ν_{33}		1461	δHCC(33) + δCCC(12)	121.44
ν_{34}		1451	δHCC(59)	143.39
ν_{35}		1447	δHCH(46) + τHCCN(12)	24.70
ν_{36}		1445	δHCH(72) + τHCOC(17)	46.28
ν_{37}	1473	1438	δHCH(76) + τHCOC(23)	10.04
ν_{38}		1428	δHCC(46) + νCC(11) in the aromatic ring	14.51
ν_{39}		1420	δHCH(81)	24.50
ν_{40}		1416	δHCH(56) + δCCC(15) + τHCCN(10)	47.39
ν_{41}		1415	δHCH(40) + δCCC(10) + τHCCN(10)	62.03
ν_{42}		1402	δHCH(44) + νNC(20) in the triazole ring + δCCC(14)+τHCCN(10)	12.68
ν_{43}	1365	1385	νNC(21) in the triazole ring + δHCC(11)	51.15
ν_{44}		1368	δHCH(83)	5.14
ν_{45}	1346	1344	δHCN(31) + νNN(15) in the triazole ring	91.01
ν_{46}	1311	1328	δHCN(27) + νNN(24) in the triazole ring	32.77
ν_{47}	1303	1299	νCC(42) in the aromatic ring + δHCC(15)	1.17
ν_{48}		1297	νCC(40) in the aromatic ring + δHCC(10)	12.67

ν_{49}		1291	νCC(68) in the aromatic ring	23.85
ν_{50}	1279	1284	νNN(37) in the triazole ring + νNC(11) in the triazole + δCNN(11)	18.01
ν_{51}		1278	δHCC(83)	8.41
ν_{52}	1279	1274	νCC(34) in the aromatic ring + δHCC(16)	2.70
ν_{53}		1249	δHCC(35)	81.38
ν_{54}		1230	νOC(41) in the methoxy group + νCC(10) in the aromatic ring	273.43
ν_{55}	1213	1223	νOC(24) in the C12—O51 + δHCN(14) + νCC(13) in the aromatic ring	405.87
ν_{56}		1211	νNC(47) in the N48-C25/N46—C24 + νCC(12) in the C24—C25	91.87
ν_{57}	1183	1197	νOC(27) in the O51—C1	161.05
ν_{58}		1183	νNC(20) in the N48—C25/N46—C24	326.66
ν_{59}	1163	1157	τHCOC(50) + δHCH(22)	8.40
ν_{60}		1149	δHCC(35)	10.31
ν_{61}	1147	1146	δHCC(52)	9.64
ν_{62}		1140	δHCC(54)	23.28
ν_{63}		1134	δHCC(68)	4.35
ν_{64}	1116	1129	νNN(20) in the N45-N46 + δHCC(14)	193.69
ν_{65}		1124	τHCOC(75) + δHCH(24)	0.65
ν_{66}		1107	νNN(10) in the N45—N46	208.60
ν_{67}	1088	1092	δHCC(45) + νCC(25) in the aromatic phenyl	12.19
ν_{68}	1071	1067	δHCC(27) + νCC(23) in the aromatic phenyl	52.94
ν_{69}	1035	1055	νNN(25) in the N49—N50 + τHCCN(10)	8.97
ν_{70}		1053	νNC(14) in the N48-C25/N46—C24	16.14
ν_{71}		1041	τHCCN(49) + δHCH(12)	6.88
ν_{72}	1020	1022	τHCCN(42) + δHCH(12)	9.95
ν_{73}		1015	νOC(52) in the O53—C41	41.17
ν_{74}	1001	1003	νCC(33) in the phenyl + δHCC(12) + δCCC(11)	9.48
ν_{75}		1000	νCC(29) in the phenyl + νNN(10) in the N49—N50	23.27
ν_{76}		978	δCCC(50) + νNN(12) in the N49—N50	8.54
ν_{77}	976	977	δCCC(55)	1.58
ν_{78}		973	δCCC(53)	1.62
ν_{79}		959	δCCC(10)	50.54
ν_{80}	964	956	τHCCC(74) + δNNN(26)	4.63
ν_{81}		955	τHCCC(70) + δNNN(12)	86.23
ν_{82}		943	τHCCC(64) + τCCCC(13)	0.19
ν_{83}		941	τHCCC(56)	0.40
ν_{84}	916	938	τHCCC(72)	0.24
ν_{85}		925	τHCNN(80)	18.74
ν_{86}		913	τHCCC(67) + τCCCN(12)	0.43
ν_{87}		889	τHCCC(55) + τCCOC(18)	5.65
ν_{88}		881	τHCCC(58)	42.46
ν_{89}		877	δOCN(20) + δNNC(11)	55.92
ν_{90}	895	866	τHCCC(79)	0.51
ν_{91}		819	τHCCC(52) + γOCCC(19)	54.95
ν_{92}		809	τHCCC(34)	30.46
ν_{93}		806	τHCCC(23)	29.08
ν_{94}		788	δCCC(12)	3.26
ν_{95}		786	τHCCC(79)	4.97
ν_{96}		771	τHCCC(47)	19.94
ν_{97}		753	τHCCC(26)	37.65

ν_{98}	747	748	γONCC(63) + γCCNC(16) + τCNNN(13)	1.82
ν_{99}	718	729	τHCCC(30)	30.42
ν_{100}		708	τHCCC(33)	0.97
ν_{101}	660	679	τCNNN(40) + τHCCC(32) + τCCCC(11)	27.26
ν_{102}		678	τCNNN(33) + τHCCC(27) + τCCCC(10)	3.45
ν_{103}		675	δCNN(12)	1.75
ν_{104}		673	γCCOC(20) + τHCCC(16)	28.96
ν_{105}	650	643	τCNNN(35) + γONCC(14)	0.12
ν_{106}	650	635	δCCC(15)	1.99
ν_{107}	625	629	τCCCC(22) + δCOC(12)	0.49
ν_{108}		615	τCCCC(20) + δCOC(10)	8.07
ν_{109}		611	δCCC(21)	32.36
ν_{110}		605	δCCC(82)	2.25
ν_{111}	592	590	τHNNC(85)	59.80
ν_{112}	574	565	δCCC(43) + δCCO(10)	4.40
ν_{113}	540	558	δCNN(13)	5.33
ν_{114}		554	τCCCC(24) + δCOC(13)	6.37
ν_{115}	520	510	γOCCC(17) + γCNCC(15)	9.13
ν_{116}	496	487	δCCN(13)	4.68
ν_{117}	478	478	τCCOC(26)	9.81
ν_{118}		473	τCCOC(21)	3.99
ν_{119}	457	445	τCCCC(28) + γCCOC(19)	5.38
ν_{120}	433	422	δOCN(12) + τCCCN(12)	6.89
ν_{121}	412	413	τCCCN(36) + τHCCC(10)	0.23
ν_{122}		408	τCCCN(30) + τHCCC(11)	6.04
ν_{123}	401	407	τCCCN(28) + τHCCC(10)	0.73
ν_{124}		393	δCCN(16) + γCCNN(11) + τCCCN(10)	1.26
ν_{125}		368	δCCO(26)	6.09
ν_{126}		342	δCCC(14)	7.96
ν_{127}		312	τCCNN(37) + τCCCC(28)	0.18
ν_{128}		293	δCCO(16)	0.29
ν_{129}		271	γCNCC(22)	0.59
ν_{130}		259	γCNCC(22)	3.08
ν_{131}		242	δCCO(18) + δOCC(18)	15.11
ν_{132}		240	τHCOC(28)	3.98
ν_{133}		237	τHCOC(14)	2.78
ν_{134}		224	τCCOC(19) + δCOC(10)	2.28
ν_{135}		211	δCCC(11)	18.46
ν_{136}		198	τCOCC(21) + τCNNC(11)	7.64
ν_{137}		193	τCOCC(24) + τCNNC(10)	0.44
ν_{138}		177	τCOCC(22) + τCNNC(13)	4.11
ν_{139}		147	τCOCC(21) + τCNNC(11)	2.56
ν_{140}		121	τCCCC(24) + τCNNC(22)	0.63
ν_{141}		109	τHCCN(70)	0.44
ν_{142}		102	τCOCC(43)	4.07
ν_{143}		84	τCOCC(38)	0.46
ν_{144}		79	δNCC(12) + δCCN(10)	2.95
ν_{145}		61	δNCC(11) + δCCN(10)	7.10
ν_{146}		54	δNCC(10) + δCCN(10)	0.17
ν_{147}		44	τCCCN(14)	0.19
ν_{148}		39	τCCCN(10)	0.96

ν_{149}	33	τCCOC(37)	0.74
ν_{150}	23	δCCN(13) + τCCCN(11)	1.02
ν_{151}	16	δCCN(11) + τCCCN(10)	1.79
ν_{152}	13	δCCN(13) + τCCCN(11)	0.30
ν_{153}	9	δCCN(12) + τCCCN(10)	0.48

[a] Assignments are made based on the results B3LYP and potential energy distribution (PED), less than 10% are not shown. R^2 values were computed as *0.9079* for IR wavenumber. ν, stretching; δ, in-plane bending; τ, torsion; γ, out-of-plane bending.
