# Supplementary figures and images for: Quantum Computational Investigation of (E)-1-(4-methoxyphenyl)-5-methyl-N′-(3-phenoxybenzylidene)-1H-1,2,3-triazole-4-carbohydrazide
Source: Molecules. 2022 Mar 28;27(7):2193. doi: 10.3390/molecules27072193 (PMC9000758; doi:10.3390/molecules27072193)

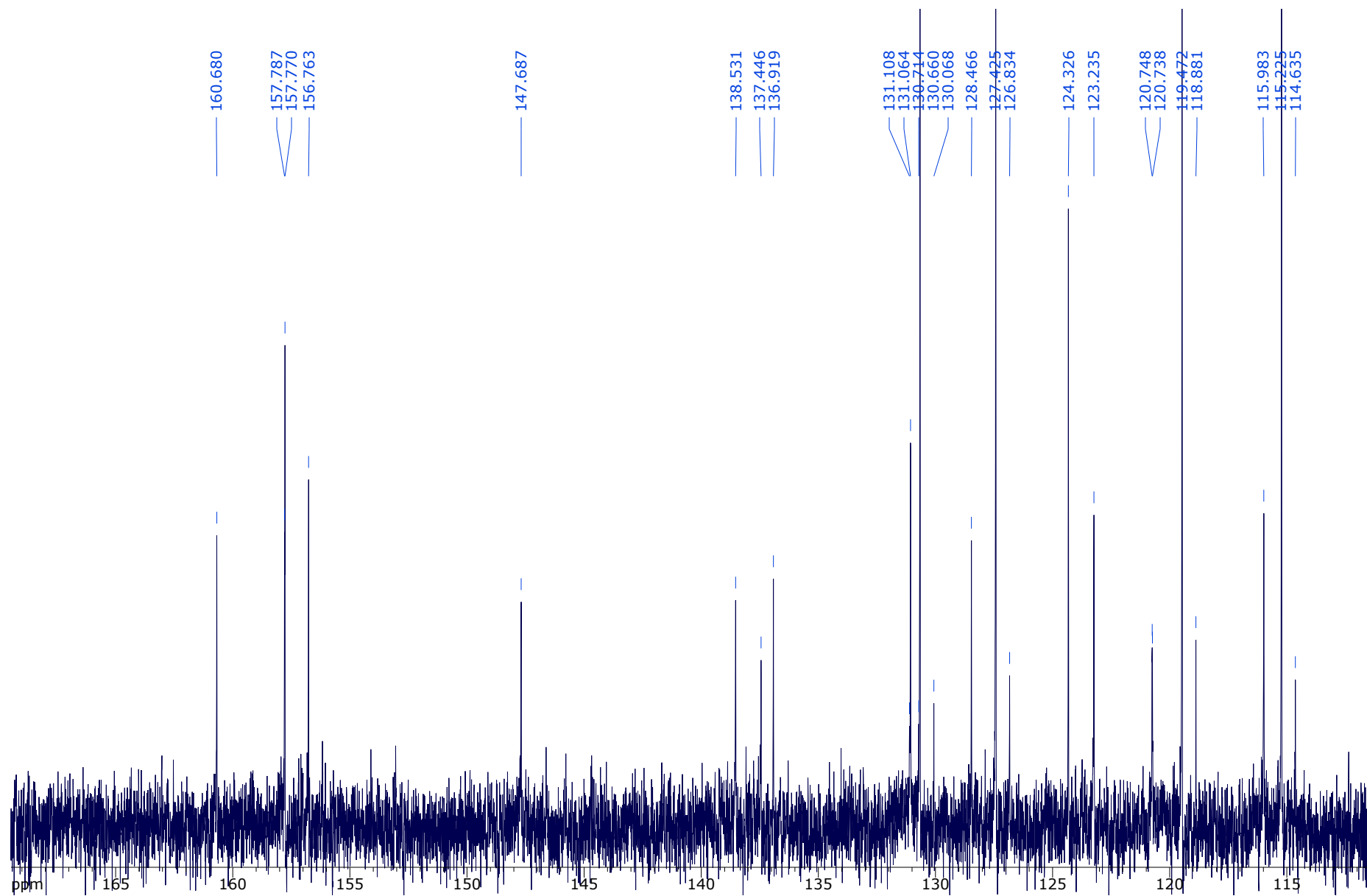

Supplement: Supplementary file 1 [file molecules-27-02193-s001.zip › 13C NMR Exp.pdf]

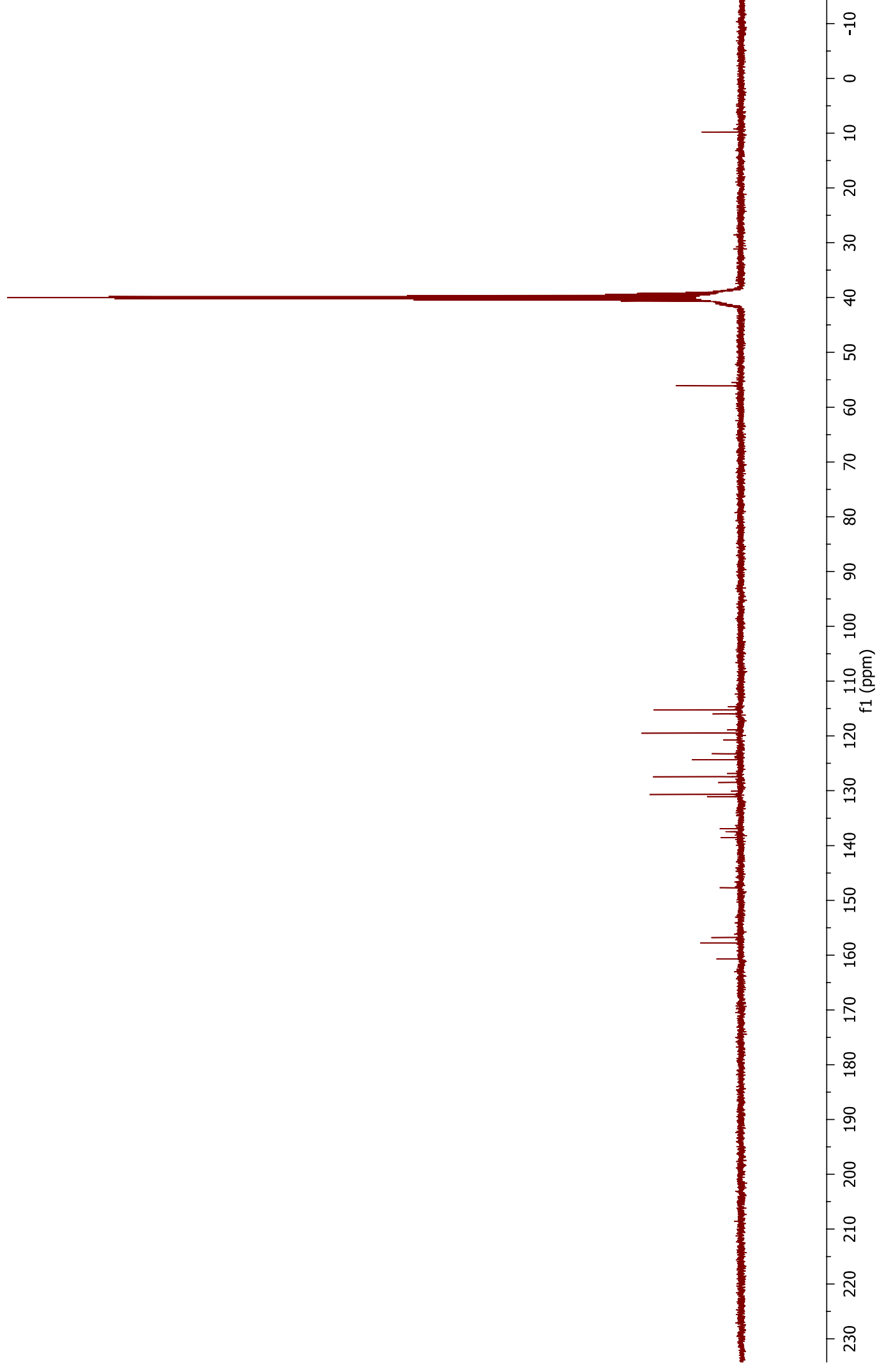

Supplement: Supplementary file 1 [file molecules-27-02193-s001.zip › 13C NMR.pdf]

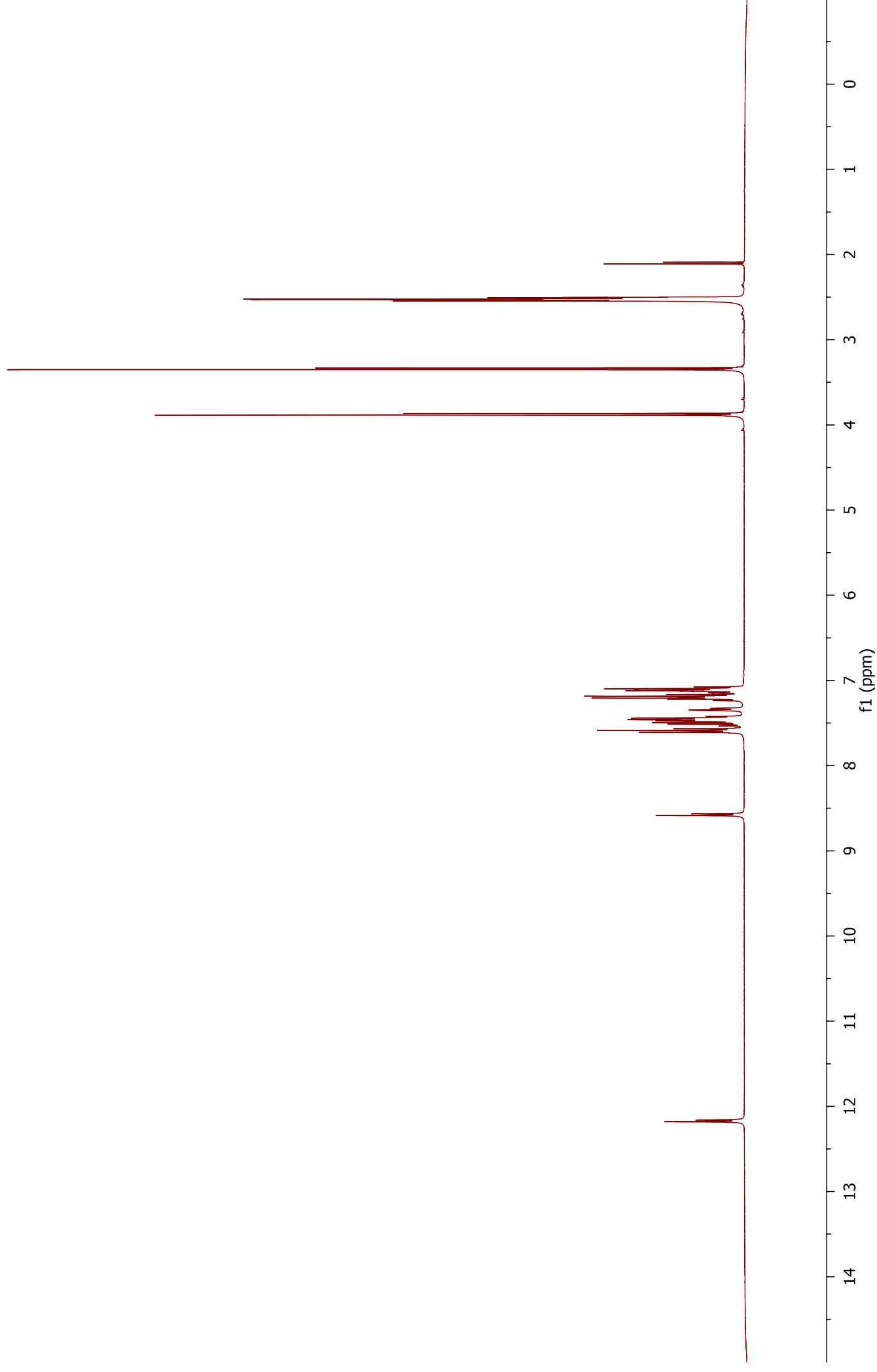

Supplement: Supplementary file 1 [file molecules-27-02193-s001.zip › 1H NMR 1.pdf]

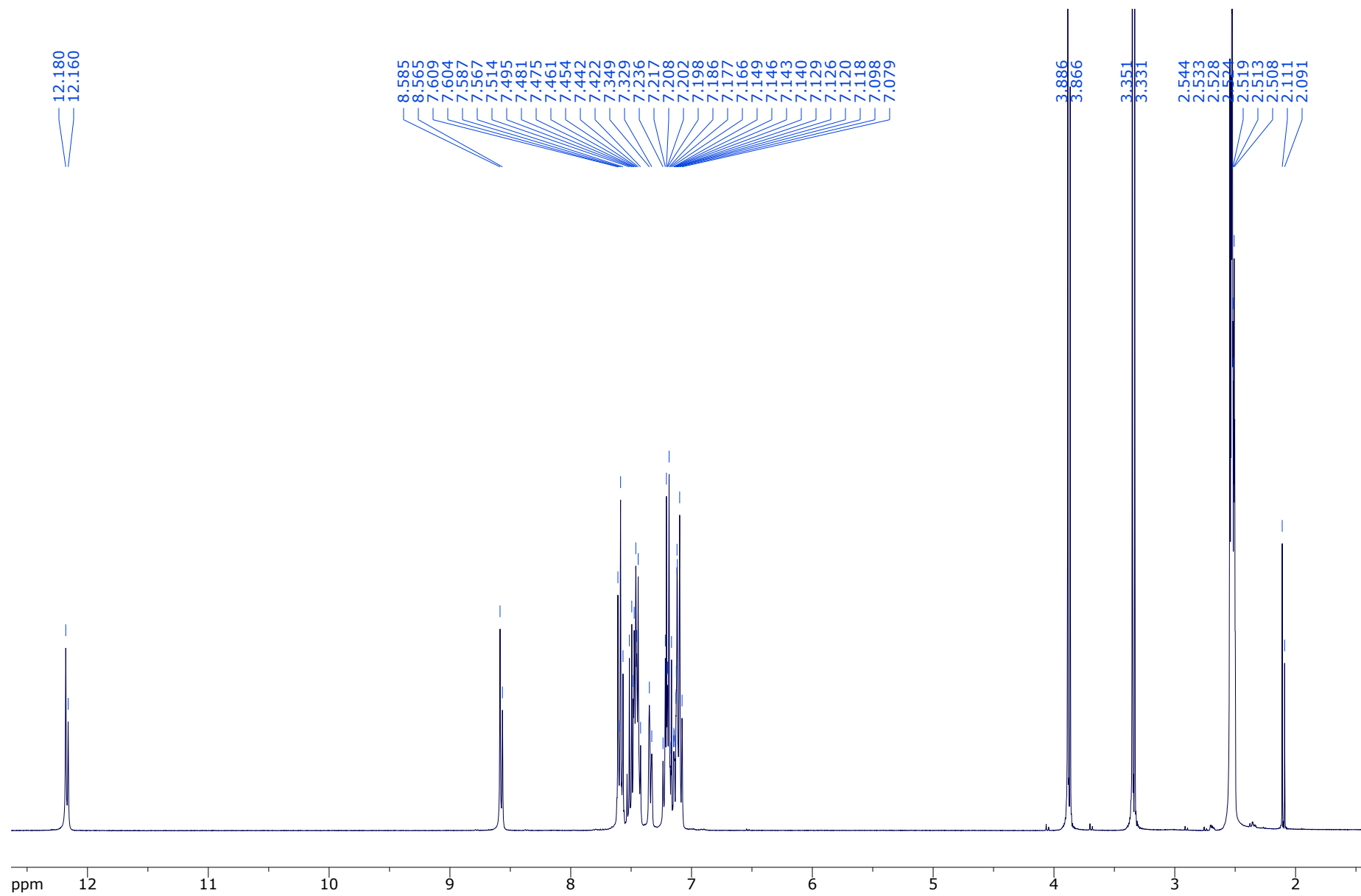

Supplement: Supplementary file 1 [file molecules-27-02193-s001.zip › 1H NMR 2.pdf]

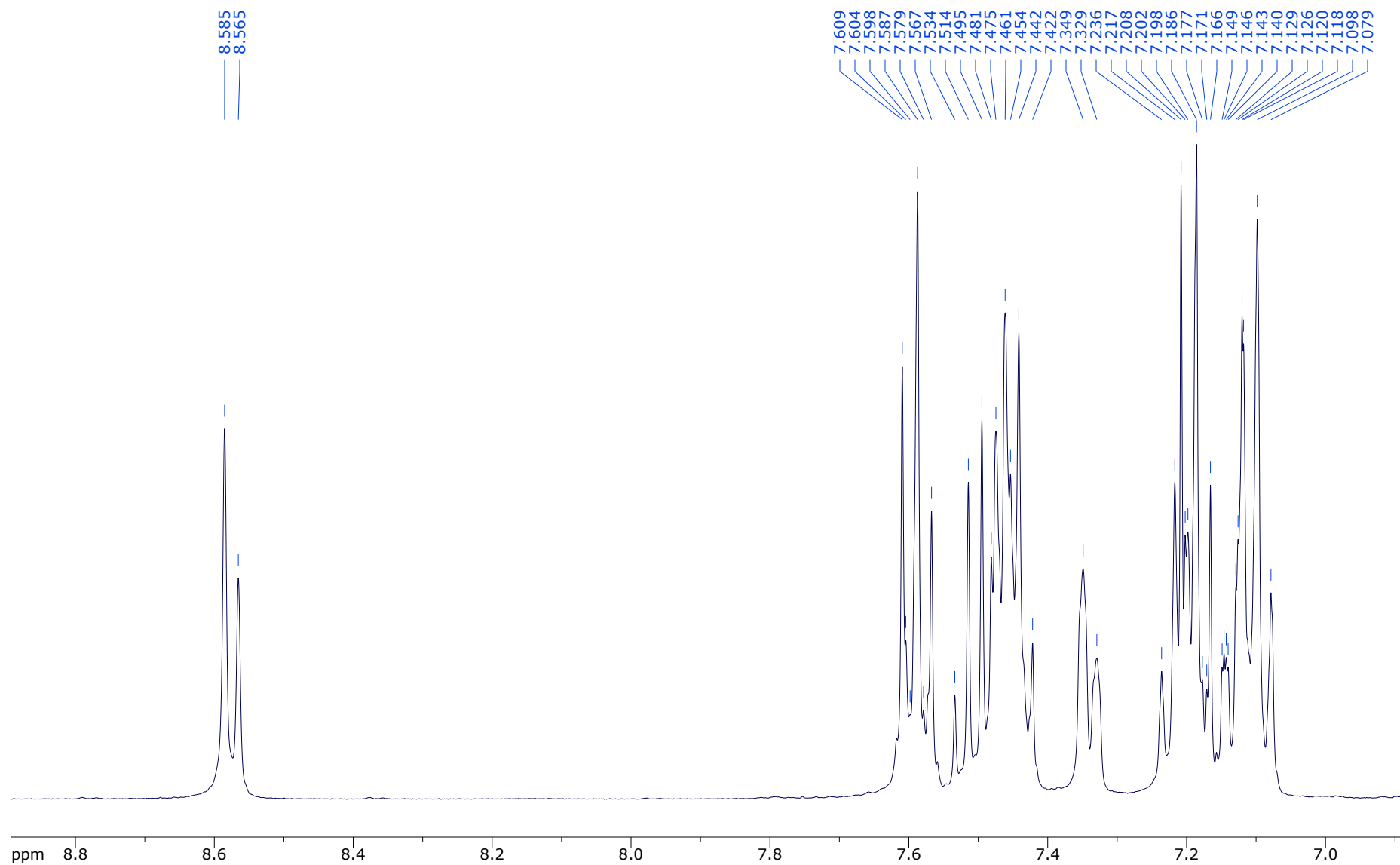

Supplement: Supplementary file 1 [file molecules-27-02193-s001.zip › 1H NMR Exp.pdf]

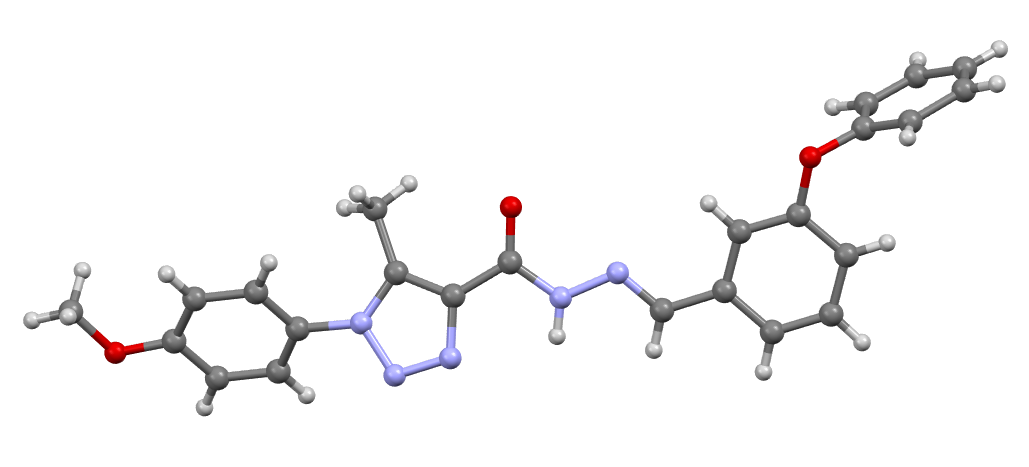

Supplement: Supplementary file 1 [file molecules-27-02193-s001.zip › bmk2003e.tif]
